# Role of Sex-Concordant Gene Expression in the Coevolution of Exaggerated Male and Female Genitalia in a Beetle Group

**DOI:** 10.1093/molbev/msab122

**Published:** 2021-04-27

**Authors:** Shota Nomura, Tomochika Fujisawa, Teiji Sota

**Affiliations:** 1 Department of Zoology, Graduate School of Science, Kyoto University, Sakyo, Kyoto, Japan; 2 The Center for Data Science Education and Research, Shiga University, Hikone, Shiga, Japan

**Keywords:** character evolution, interspecific differences, sexual traits, transcriptome, weighted gene co-expression network analysis

## Abstract

Some sexual traits, including genitalia, have undergone coevolutionary diversification toward exaggerated states in both sexes among closely related species, but the underlying genetic mechanisms that allow correlated character evolution between the sexes are poorly understood. Here, we studied interspecific differences in gene expression timing profiles involved in the correlated evolution of corresponding male and female genital parts in three species of ground beetle in *Carabus* (*Ohomopterus*). The male and female genital parts maintain morphological matching, whereas large interspecific variation in genital part size has occurred in the genital coevolution between the sexes toward exaggeration. We analyzed differences in gene expression involved in the interspecific differences in genital morphology using whole transcriptome data from genital tissues during genital morphogenesis. We found that the gene expression variance attributed to sex was negligible for the majority of differentially expressed genes, thus exhibiting sex-concordant expression, although large variances were attributed to stage and species differences. For each sex, we obtained co-expression gene networks and hub genes from differentially expressed genes between species that might be involved in interspecific differences in genital morphology. These gene networks were common to both sexes, and both sex-discordant and sex-concordant gene expression were likely involved in species-specific genital morphology. In particular, the gene expression related to exaggerated genital size showed no significant intersexual differences, implying that the genital sizes in both sexes are controlled by the same gene network with sex-concordant expression patterns, thereby facilitating the coevolution of exaggerated genitalia between the sexes while maintaining intersexual matching.

## Introduction

Correlated evolution or the coevolution of sexual traits between the sexes among closely related species, often toward exaggerated character states, is notable in some animal lineages and has long attracted the interest of evolutionary biologists (Fisher 1930; Lande 1980, 1981; Panhuis et al. 2001; Mead and Arnold 2004). The coevolutionary dynamics of corresponding male and female characters are complex, depending on the type of selection (sex-concordant or discordant) and genetic covariation of male and female characters (Lande 1980, 1981; Mead and Arnold 2004), and the genetic mechanisms underlying coevolution between the sexes are poorly understood.

The genitalia of animals with internal fertilization are representative sexual characters that show correlated evolution between the sexes toward exaggerated states in some lineages (Brennan and Prum 2015; Langerhans et al. 2016). Genital coevolution can be caused by natural selection to resolve a problem common to both sexes, such as the avoidance of hybridization or predation, female choice, and sexual conflict (Brennan and Prum 2015; Langerhans et al. 2016). In addition, pleiotropy or a shared genetic/developmental basis for the genitalia of both sexes may facilitate coevolution between the sexes (Langerhans et al. 2016). Because male and female genitalia require morphological matching to achieve efficient insemination, selection would operate to prevent excessive departure from matching during coevolution; alternatively, genetic correlations in the size and shape of genitalia between the sexes may maintain the matching. Indeed, a quantitative genetic study of a dung beetle species found a positive intersexual genetic correlation in genital size and covariation in genital shape (Simmons and Garcia-Gonzalez 2011). For homologous characters in both sexes, such as body size, a positive intersexual genetic correlation would allow rapid coevolution in the same direction, although it impedes sexual divergence under sex-discordant selection (Lande 1980; Poissant et al. 2010; Stewart and Rice 2018). Positive genetic correlations between the sexes may be based on similar gene expression involved in the development of the homologous characters. Although male and female genitalia may not be completely homologous (e.g., male and female genitalia derive from different segments and tissues in insects; Sánchez and Guerrero 2001), the genital sizes of both sexes may be controlled by gene expression patterns shared between the sexes. Meanwhile, interspecific differences in genital morphology in both sexes would be based on species-specific gene expression, and these two mechanisms may have enabled genital coevolution with matching between the sexes.

**Fig. 1.**
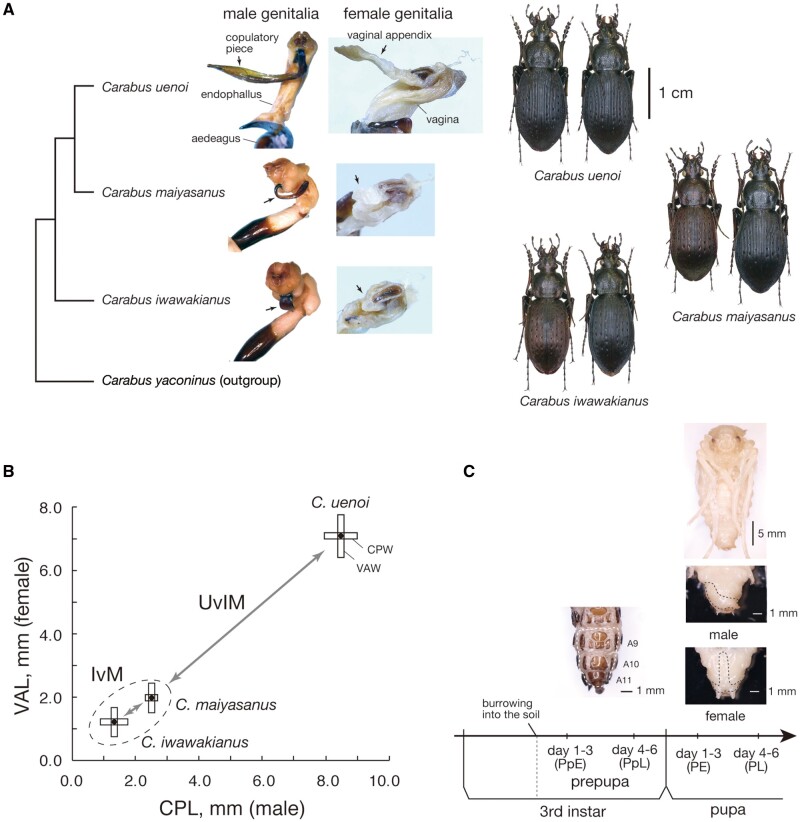
The species of *Carabus* (*Ohomopterus*) studied. (*A*) Phylogenic relationships of *Carabus iwawakianus*, *C. maiyasanus*, and *C*. *uenoi* with the outgroup *C. yaconinus* (Fujisawa et al. 2019). Photographs of male and female genitalia and male (left) and female (right) beetles are shown. (*B*) Interspecific differences and matching between the sexes of genital part sizes in *C*. *iwawakianus*, *C*. *maiyasanus*, and *C*. *uenoi* (data from Sasabe et al. 2010). Black dots indicate copulatory piece length (CPL) and vaginal appendix length (VAL); horizontal and vertical bars indicate copulatory piece width (CPW) and vaginal appendix width (VAW), respectively. IvM denotes the comparison between *C*. *iwawakianus* and *C*. *maiyasanus* and UvIM the comparison between *C*. *uenoi* and *C*. *iwawakianus*/*C*. *maiyasanus*. (*C*) Developmental stages and timing of RNAlater fixation in the third instar and pupal stages. Abdominal parts of a third instar larva, and male and female pupae are shown to indicate the dissected portions for RNA extraction.

The ground beetles in the subgenus *Ohomopterus* (genus *Carabus*) endemic to Japan exhibit notable coevolution between male and female genital parts (copulatory piece and vaginal appendix) toward exaggeration in 17 species (Sota and Nagata 2008; Sasabe et al. 2010; Fujisawa et al. 2019). During copulation, the copulatory piece is inserted into a vaginal appendix of the corresponding size and shape to secure proper genital coupling leading to insemination (Takami 2002, 2003). It is not clear how and why coevolution toward exaggerated states could occur despite natural and sexual selection for morphological matching between the male and female parts (Sota and Kubota 1998; Takami 2003; Okuzaki and Sota 2014; Takami et al. 2018). The genetic background of species-specific male and female genital morphology has been investigated using hybridization between the sister species *Carabus iwawakianus* and *C. maiyasanus* (Sasabe et al. 2007, 2010; Fujisawa et al. 2019). These studies showed that genital part sizes are controlled by a few major quantitative trait loci (QTL) for the lengths of male and female genital parts in different regions of the same chromosome, implying that the evolution of genital part length is only weakly constrained between the sexes. These species are closely related to *C. uenoi*, which possesses the most exaggerated genitalia, and these three species together provide intriguing material for studying the genetic background of the coevolution of exaggerated genitalia (fig. 1). However, because of the extreme genital size of *C*. *uenoi*, it is impossible to perform genetic analyses using interspecific crossing involving *C*. *uenoi*. Interspecific differences in genital morphology should be based on different gene expression patterns during genital morphogenesis. Therefore, one way to study the genetic basis of exaggerated genitalia is to compare the transcriptomes of the species during genital morphogenesis. Previously, we clarified the expression timing profiles of genes related to metamorphosis and genital morphogenesis of *C*. *maiyasanus* at different stages, which provided basic information for studying interspecific differences in gene expression related to species-specific genital morphology (Nomura et al. 2020).

This study examined the genetic basis of interspecific differences in genital morphology and the coevolution of male and female genital morphology based on a comparison of gene expression profiles among *C*. *iwawakianus*, *C*. *maiyasanus*, and *C*. *uenoi* (fig. 1*A*). For simplicity, we performed two interspecific comparisons: *C*. *iwawakianus* versus *C*. *maiyasanus* (IvM) and *C*. *uenoi* versus *C*. *iwawakianus* and *C*. *maiyasanus* (UvIM) (fig. 1*B*). The IvM comparison involved distinct shape differences, but small size differences studied in previous genetic studies (Sasabe et al. 2007, 2010; Fujisawa et al. 2019), whereas the UvIM comparison involved extreme size differences, which had not been subject to genetic analyses. For each comparison, we examined differences in the gene expression profiles at four immature stages and identified differentially expressed genes (DEGs) that exhibited differences in expression levels between stages, sexes, or species. Then we obtained the candidate sets of genes related to interspecific differences in genital shape and size from the DEGs in each sex by sorting the DEGs into clusters (modules) according to their expression patterns using weighted gene co-expression network analysis (WGCNA; Langfelder and Horvath 2008). To infer how coevolution between male and female genitalia could be achieved in each comparison, we investigated the expression profiles of central hub genes, which presumably represented average expression patterns of the candidate gene networks across species, stages, and sexes. We found that the sex-discordant expression of shared gene coexpression networks drove the coevolution of species-specific genital shapes with little size difference (the IvM comparison), whereas sex-concordant expression profiles in gene coexpression networks commonly involved in the genital morphogenesis of both sexes underlie the coevolution of exaggerated genitalia between the sexes (the UvIM comparison). Our results indicate that, during the coevolution of exaggerated genitalia, a regulatory change in genital size shared by both sexes may have played a major role.

## Results

### Transcriptome Differences between Stages, Sexes, and Species

We obtained transcriptomic mRNA sequence data for both sexes of *C*. *maiyasanus*, *C*. *iwawakianus*, and *C*. *uenoi* from abdominal tissues of four developmental stages in third instar larvae (prepupal stage: days 1–3, PpE; days 4–6, PpL) and pupae (days 1–3: PE; days 4–6, PL; fig. 1*C*; supplementary table S1, Supplementary Material online). We mapped all sequence reads to the reference *C*. *uenoi* genome sequence with similar mapping rates for all samples and obtained expression data for 18,839 genes. Expression variation analysis of all genes showed that the mean percentages of variance explained by species and stage were 13.8% (max, 91.8%) and 17.7% (max, 75.9%), respectively, whereas that explained by sex was only 1.8 × 10^−2^% (max, 61.1%) (supplementary fig. S1, Supplementary Material online). Principal component analysis (PCA) of the expression timing profiles (hereafter expression patterns) of all genes revealed that individual samples of the same stages and species clustered irrespectively of sex (supplementary fig. S2, Supplementary Material online), and there were significant differences in principal component scores 1 (PC1) and PC2 among species and stages, but no difference was found between the sexes (supplementary table S2, Supplementary Material online). These results showed that the effect of sex on the total variation in gene expression was, on average, extremely small compared with the effects of species and stage.

### DEGs between Species

**Fig. 2.**
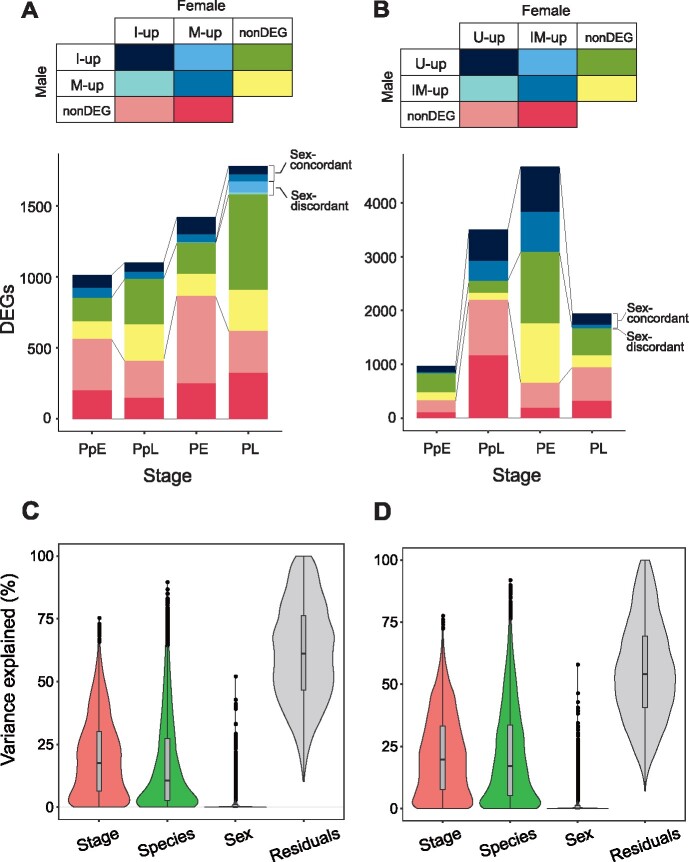
(A, *B*) Comparison of interspecific DEGs in males and females at each developmental stage. (*A*) IvM comparison (between *Carabus iwawakianus* and *C. maiyasanus*). For each sex, I-up and M-up indicate up-regulation in *C*. *iwawakianus* and *C*. *maiyasanus*, respectively; nonDEG, not differentially expressed between the species. (*B*) UvIM comparison (between *C. uenoi* and *C*. *iwawakianus*/*C*. *maiyasanus*). For each sex, U-up indicates up-regulation in *C*. *uenoi* versus both *C*. *iwawakianus* and *C*. *maiyasanus*, and IM-up indicates up-regulation in both *C*. *iwawakianus* and *C*. *maiyasanus* versus *C*. *uenoi*; nonDEG, not differentially expressed between the species between *C*. *uenoi* and *C*. *iwawakianus*/*C*. *maiyasanus* or oppositely regulated between *C*. *uenoi* versus *C*. *iwawakianus* and *C*. *uenoi* versus *C*. *maiyasanus* comparisons. (*C*, *D*) Violin plots showing the distribution of percentages of the expression variance explained by stage, species, and sex differences for 3,895 DEGs in the IvM comparison (*C*) and for 7,031 DEGs in the UvIM comparison (*D*).

To investigate differences in gene expression among species that may be related to differences in genital morphology, we determined DEGs that showed differences between species at any developmental stage and sex at a false discovery rate (FDR) < 0.05 in the *C*. *iwawakianus* versus *C*. *maiyasanus* (IvM) and *C*. *uenoi* versus *C*. *iwawakianus*/*C*. *maiyasanus* (UvIM) comparisons and found 3,895 and 7,031 DEGs for the respective comparisons (fig. 2*A* and *B*). In the IvM comparison, many of the DEGs between species were differentially expressed only in one sex; among genes differentially expressed in both sexes, 160, 118, 179, and 110 genes showed sex-concordant regulation (i.e., up or downregulated in both sexes) in the PpE, PpL, PE, and PL stages, respectively, whereas 0, 0, 6, and 94 genes showed sex-discordant regulation in the respective stages (fig. 2*A*). In the UvIM comparison, where we focused on DEGs in both the *C. uenoi* versus *C. iwawakianus* and *C. uenoi* versus *C. maiyasanus* comparisons, we found relatively high numbers of DEGs showing sex-concordant regulation (141, 955, 1,592, and 280 genes in the PpE, PpL, PE, and PL stages, respectively) but very few DEGs with sex-discordant regulation (0, 1, 1, and 2 genes in the PpE, PpL, PE, and PL stages, respectively; fig. 2*B*).

Expression variation analysis of DEGs revealed that the mean percentages of variance explained by species and stage were 17.3% (max, 89.6%) and 19.9% (max, 75.2%) in the IvM comparison and 21.6% (max, 91.8%) and 21.7% (max, 77.5%) in the UvIM comparison (fig. 2*C* and *D*). The mean percentages of variance explained by sex were relatively small, 1.33% (max, 52.0%) in the IvM comparison and 1.37% (max, 57.9%) in the UvIM comparison. Some of the DEGs showing large expression variance between the sexes (>5%, arbitrary) were involved in imaginal disc development: 20 of 283 and 50 of 541 genes in the IvM and UvIM comparisons, respectively, with significant enrichment in the latter comparison (supplementary tables S3 and S4, Supplementary Material online). Some of the imaginal disc genes were involved in genital morphogenesis, such as *doublesex* (*dsx*), *abdominal A* (*Abd-A*), *thickvein* (*tkv*), *Lim homeobox 1* (*Lim1*), *rotund* (*rn*).

Notably, there were many interspecific DEGs that were differentially expressed in only one sex at each developmental stage (fig. 2*A* and *B*), which seemingly contradicted the relatively small variance explained by the sex difference (fig. 2*C* and *D*). However, the expression variances were partitioned according to the overall gene expression data including the effects of stage, species, and sex, whereas the categorization of interspecific DEGs at each stage only considered the interspecific expression difference in each sex without accounting for the magnitude of differential expression between sexes.

### Weighted Co-Expression Gene Network Analysis

To identify genes that might be involved in differences in genital morphology, we clustered the DEGs according to the similarity of expression patterns in IvM and UvIM using WGCNA. Because male and female genitalia are not strictly homologous and may be formed as a result of sex-specific interactions among genes, we clustered the DEGs in each sex. In the IvM comparison, the DEGs clustered into 13 modules comprising 66–599 genes in males, and into 12 modules comprising 123–708 genes in females (supplementary table S5, Supplementary Material online). In the UvIM comparison, the DEGs clustered into 11 modules comprising 101–1,637 genes in males, and into 12 modules comprising 100–1,057 genes in females (supplementary table S6, Supplementary Material online). We performed GO enrichment analysis of these modules to find modules enriched with functions related to genital development. Among the GO terms, we focused on “imaginal disc” terms related to wing development or leg morphogenesis, which shared genes with genital morphogenesis in *Drosophila* (Sánchez and Guerrero 2001), and “cuticle development” which may also be related to the species-specific genital morphology in *Ohomopterus* (Fujisawa et al. 2019). Thus, we assumed that genes involved in “imaginal disc-derived appendage development,” “imaginal disc-derived wing morphogenesis,” and “cuticle development” were also involved in genital development.

In the IvM comparison, male module M5 and female modules F4 and F6 were enriched with GO terms for appendage development, whereas male module M2 and female module F2 were enriched with the GO term for cuticle development (FDR < 0.01; table 1). In the UvIM comparison, male modules M1, M5, and M10 and female modules F2, F6, and F7 were enriched with GO terms for appendage development or wing morphogenesis, and male module M3 and female module F3 were enriched with the GO term for cuticle development (FDR < 0.01; table 2).

**Table 1.** Number of Genes Included in GO Terms Putatively Related to Genital Morphogenesis in IvM Modules.

	IvM Male Module (*C. iwawakianus* vs. *C*. *maiyasanus*)												
GO term	M1	M2*	M3	M4	M5*	M6	M7	M8*	M9	M10	M11	M12	M13
Imaginal disc-derived appendage development	0	0	0	0	20∗	0	0	0	0	0	0	0	0
Imaginal disc-derived wing morphogenesis	0	0	0	0	16∗	0	0	0	0	0	0	0	0
Hippo signaling pathway—fly	0	0	0	0	3	0	0	0	0	0	0	0	0
Cuticle development	0	21∗	0	0	0	0	0	0	0	0	0	0	0
Developmental pigmentation	0	0	0	0	0	0	0	0	0	0	0	0	0

	IvM Female Module (*C. iwawakianus* vs. *C. maiyasanus*)													
GO term	F1	F2*	F3	F4*	F5	F6*	F7	F8	F9	F10	F11	F12		
Imaginal disc-derived appendage development	0	0	0	20∗	0	18∗	0	0	0	0	0	0		
Imaginal disc-derived wing morphogenesis	0	0	0	16	0	12	0	0	0	0	0	0		
Hippo signaling pathway—fly	6	0	0	0	0	0	0	0	0	0	0	0		
Cuticle development	0	16∗	0	11	0	0	0	0	7	0	0	0		
Developmental pigmentation	0	0	0	0	0	0	0	0	4	3	0	0		

Asterisks for modules indicate candidate modules for species-specific genital morphogenesis, which contained enriched GO terms and/or other genes related to genital morphogenesis.

Note.—Asterisks for numbers of genes indicate that the terms are significantly enriched in the modules (FDR < 0.01).

**Table 2.** Number of Genes Associated with GO Terms Related to Genital Morphogenesis in UvIM Modules.

	UvIM Male Module (*C. uenoi* vs. *C. iwawakianus* and *C.* *uenoi* vs. *C. maiyasanus*)											
GO term	M1*	M2	M3*	M4	M5*	M6	M7	M8	M9	M10*	M11	
Imaginal disc-derived appendage development	65∗	24	0	0	20∗	0	0	0	0	23∗	0	
Imaginal disc-derived wing morphogenesis	53∗	19	0	0	17	0	0	0	0	18∗	0	
Hippo signaling pathway—fly	11	7	0	0	8∗	0	0	0	0	0	0	
Cuticle development	0	0	26∗	0	0	0	0	0	6	0	0	
Developmental pigmentation	0	0	0	0	0	0	0	0	0	0	0	

	UvIM Female Module (*C. uenoi* vs. *C. iwawakianus* and *C.* *uenoi* vs. *C. maiyasanus*)											
GO term	F1	F2*	F3*	F4	F5	F6*	F7*	F8	F9	F10	F11	F12
Imaginal disc-derived appendage development	0	29∗	0	0	0	37∗	23∗	0	0	0	0	0
Imaginal disc-derived wing morphogenesis	0	22	0	0	0	29∗	19∗	0	0	0	0	0
Hippo signaling pathway—fly	4	8	6	0	0	0	0	0	0	0	0	0
Cuticle development	0	0	18∗	0	0	0	13	0	0	0	0	0
Developmental pigmentation	0	0	0	0	0	0	0	0	0	0	0	0

Asterisks for modules indicate candidate modules for species-specific genital morphogenesis, which contained enriched GO terms and/or contained other genes related to genital morphogenesis.

Note.—Asterisks for numbers of genes indicate that the terms are significantly enriched in the modules (FDR < 0.01).

In the UvIM comparison, we also found that genes involved in the genetic pathways likely to be involved in the formation of species-specific genital morphology were enriched in the male modules. Thus, genes in the mTOR and hedgehog signaling pathways were significantly enriched in male module M1, as well as genes in the hippo signaling pathway in male module M5 (table 3). We also determined which modules contained genes involved in organ size control. In the IvM comparison, male module M5 contained the *rn* gene, M8 contained the *dac* and *Lim1* genes, and female module F6 contained the genes *dac*, *Lim1*, and *rn*. In the UvIM comparison, male module M1 contained *Lim1* and *rn*, and M10 contained *al* and *dsx*; female module F2 contained *Abd-B*, and F7 contained *al*, *Lim1*, and *dsx*. These modules may be involved in the formation of species-specific genital morphology.

**Table 3.** Results of KEGG Pathway Analysis for DEGs Included in Each Module of the UvIM Comparison.

KEGG ID	Term	Counts	log(FDR-*P*)
UvIM Male Module M1			
dme04120	Ubiquitin mediated proteolysis	26	–5.64
dme04150	mTOR signaling pathway	18	–2.10
dme03018	RNA degradation	15	–3.10
dme04341	Hedgehog signaling pathway	11	–2.61
dme04140	Autophagy	18	–2.15
UvIM Male Module M5			
dme04391	Hippo signaling pathway	8	–2.02

Note.—Only significant terms (log[FDR-*P*] < −2) are shown.

Our results indicate that genes involved in the formation of species-specific genital morphology were contained in male modules M2, M5, and M8 and female modules F2, F4, and F6 in the IvM comparison, and male modules M1, M3, M5, and M10 and female modules F2, F3, F6, and F7 in the UvIM comparison. Therefore, we focused on these candidate modules for species-specific genital morphology in the following analyses.

### Hub Genes and Their Expression Patterns

To identify genes that primarily affect the species-specific genital morphology and to estimate their average gene expression pattern in each of the modules selected in the previous section, we identified hub genes that interact with many genes and would play a central role in the network, based on the module membership (MM; connectivity in a WGCNA module; supplementary tables S7 and S8, Supplementary Material online). We considered the 50 genes with the highest MM values for each module as hub genes, and regarded the gene with the highest MM value as the representative hub gene (hereafter, top hub gene). The top hub gene was assumed to show the typical expression profile of the module. To examine whether the genes in the modules were involved in male and female genital formations, we compared the expression levels of the top hub genes across stages, sexes, and species.

**Fig. 3.**
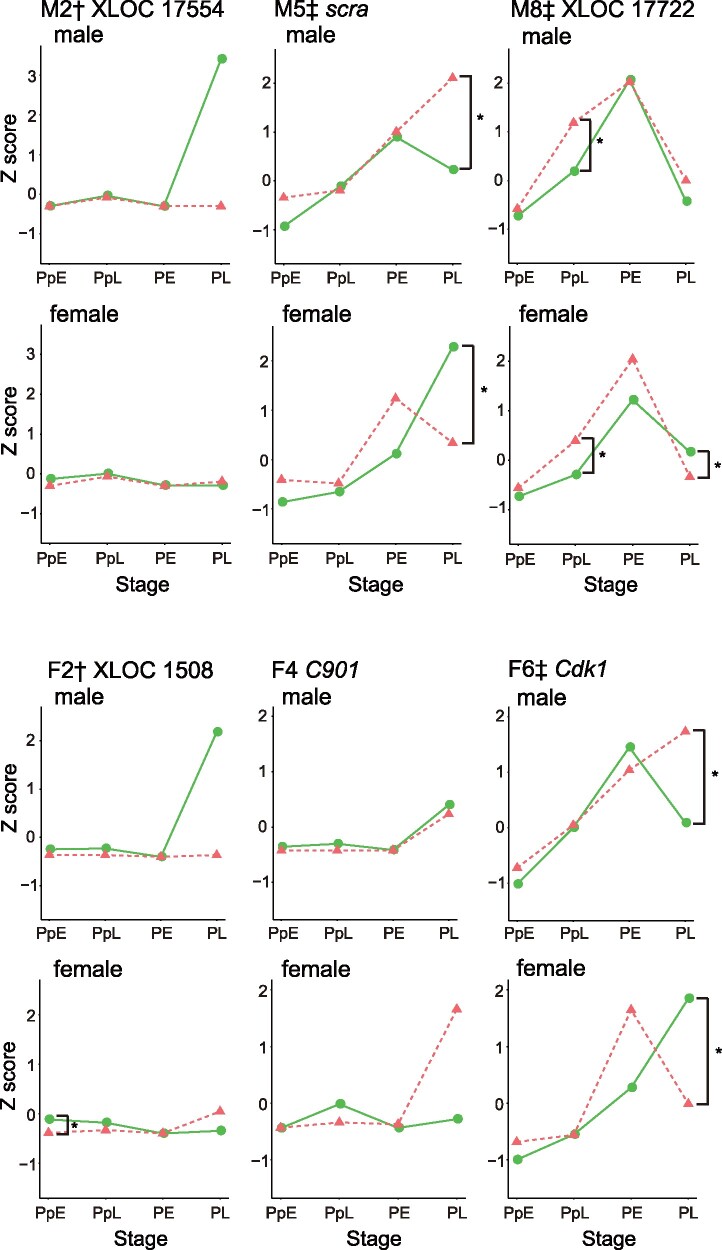
The expression profiles of the top hub genes in male and female modules of the IvM comparison. For each top hub gene, expression profiles in both sexes are shown. Male modules (top hub gene in parentheses): M2 (XLOC 17554), M5 (*scra*), and M8 (XLOC 17722); female modules: F2 (XLOC 1508), F4 (*C901*), and F6 (*Cdk1*). Green and red lines indicate the expression profiles of *Carabus iwawakianus* and *C. maiyasanus*, respectively. Asterisks indicate significantly different expression levels between species at each stage (*P *>* *0.05). Modules with the same daggers (†) or double daggers (‡) share hub genes and are common modules between the sexes.

Among the six candidate modules in the IvM comparison, male module M2 and female module F2 shared 8 hub genes, male module M5 and female module F6 shared 21 hub genes, and male module M8 and female module F6 shared 6 hub genes. These pairs of male and female modules were considered to contain common gene networks. The top hub genes were XLOC 17554, *scra* and XLOC 17722 in male modules M2, M5, and M8, respectively, and XLOC 1508, *C901*, and *Cdk1* in female modules F2, F4, and F6, respectively (note that XLOC means unannotated gene locus). The expression patterns of *scra* and *Cdk1* showed interspecific differences at the PL stage in both sexes (fig. 3). At the PL stage, *C*. *maiyasanus* expressed more *scra* and *Cdk1* in males, whereas *C*. *iwawakianus* did so in females. Thus, the expression pattern of these genes showed an interaction effect between species, stage, and sex (table 4). Although not significant, similar interspecific differences in expression patterns at the PL stage were found in XLOC 17554, XLOC 17722, XLOC 1508, and *C901* (fig. 3). However, XLOC 17722 was expressed more in both sexes of *C. maiyasanus* than in *C. iwawakianus* at the PpL stage.

**Table 4.** Effects of Stage, Sex, and Species on the Expression Levels of the Hub Genes in IvM Male Modules M2 (XLOC 17554), M5 (*scra*), and M8 (XLOC 17722) and Female Modules F2 (XLOC 1508), F4 (*C901*), and F6 (*Cdk1*) in *Carabus iwawakianus* and *C. maiyasanus*.

Factor	Df	*χ* ^2^	*P*-Value	*χ* ^2^	*P*-Value	*χ* ^2^	*P*-Value
Male module		M2 (XLOC 17554)		M5 (*scra****)***		M8 (XLOC 17722)	
Stage	3	12.576	*0.0056*	81.660	*<0.0001*	118.772	*<0.0001*
Sex	1	4.099	0.0429	1.620	0.2031	7.329	*0.0068*
Species	1	5.531	*0.0187*	6.832	*0.0090*	14.822	*0.0001*
Stage*Sex	3	13.382	*0.0039*	4.040	0.2572	12.768	*0.0052*
Stage*Species	3	12.376	*0.0062*	6.543	0.0880	13.939	*0.0030*
Sex*Species	1	4.695	*0.0303*	9.024	*0.0027*	0.284	0.5939
Stage*Sex*Species	3	14.772	*0.0020*	45.593	*<0.0001*	13.646	*0.0034*
Female module		F2 (XLOC 1508)		F4 (*C901*)		F6 (*Cdk1*)	
Stage	3	15.263	*0.0016*	25.645	*<0.0001*	79.110	*<0.0001*
Sex	1	2.625	0.1052	1.001	0.3170	3.064	0.0800
Species	1	5.620	*0.0178*	1.577	0.2092	2.257	0.1330
Stage*Sex	3	9.679	*0.0215*	1.508	0.6805	4.459	0.2160
Stage*Species	3	7.991	*0.0462*	9.603	*0.0223*	3.921	0.2701
Sex*Species	1	5.421	*0.0199*	3.695	0.0546	3.516	0.0608
Stage*Sex*Species	3	16.900	*0.0007*	11.142	*0.0110*	43.114	*<0.0001*

Italicized *P*-values are < 0.05.

Note.—The top hub genes are indicated in the parentheses.

**Fig. 4.**
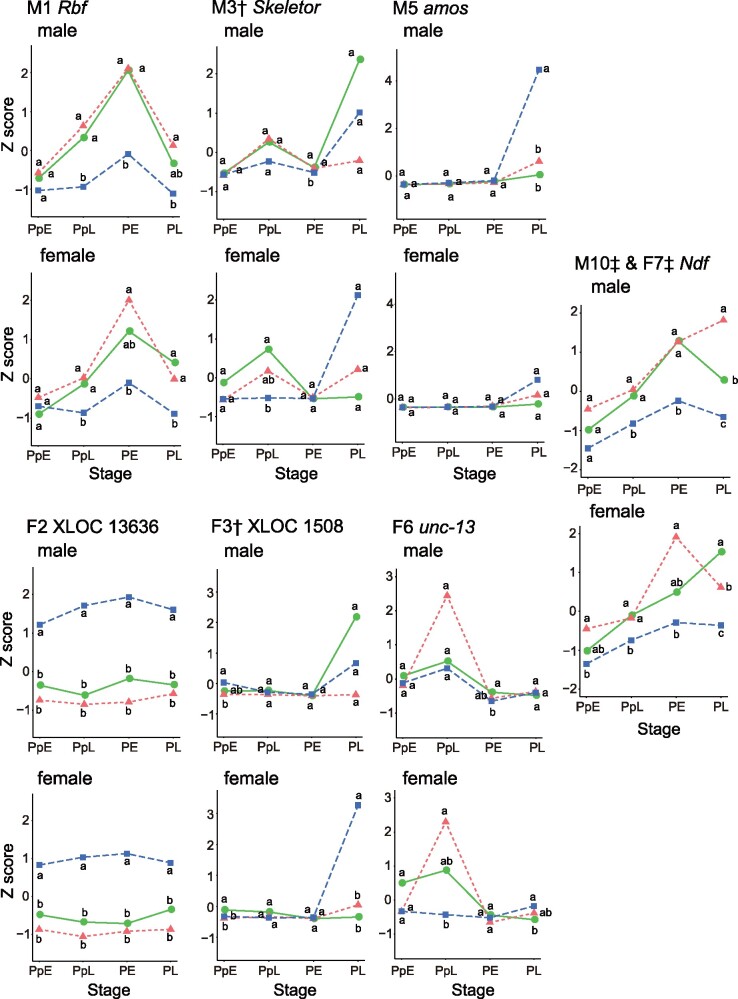
The expression profiles of the top hub genes in male and female modules of the UvIM comparison. For each top hub gene, expression profiles of both sexes are shown. Male modules (top hub gene in parentheses): M1 (*Rbf*), M3 (*Skeletor*) and M5 (*amos*), and M10 (*Ndf*); female module: F2 (XLOC 13636), F3 (XLOC 1508) and F6 (*unc-13*), and F7 (*Ndf*). Note that F7 shares the same top hub gene with M10. Green, red, and blue lines indicate the expression profiles of *Carabus iwawakianus*, *C. maiyasanus*, and *C. uenoi*, respectively. Expression levels with the same letter (a, b) are not significantly different from one another (*P *>* *0.05) among species at each stage by the multiple comparison test. Modules with the same daggers (†) or double daggers (‡) share hub genes and represent common modules between the sexes.

Among the eight candidate modules in the UvIM comparison, male module M3 and female module F3 shared 10 hub genes, and male module M10 and female module F7 shared 19 hub genes. These pairs of male and female modules were considered to contain common gene networks. Among the hub genes in each module, the top hub genes were *Rbf*, *Skeletor*, *amos*, and *Ndf* in male modules M1, M3, M5, and M10, respectively, and XLOC 13636, XLOC 1508, *unc-13*, and *Ndf* in female modules F2, F3, F6, and F7, respectively. Note that *Ndf* is the top hub gene in both male M10 and female F7 modules. Of these top hub genes, the expression of *amos* and XLOC 1508 showed interspecific differences at the PL stage in both sexes (fig. 4). The expression patterns of *amos* and XLOC 1508 showed an effect of sex and interaction effect of stage and sex (table 5), as the expression of these genes at the PL stage differed between sexes in *C. uenoi* (fig. 4). *Rbf* and *Ndf* had lower expression levels in *C. uenoi* than in the other species at all stages except for PpE in both sexes (fig. 4), and the expression pattern of these genes showed no effect of sex (table 5). The unannotated gene XLOC 13636 was expressed at higher levels in *C. uenoi* than in the other species at all stages in both sexes, and in *C. uenoi*, males expressed it more than did females (fig. 4).

**Table 5.** Effects of Stage and Sex on the Expression Levels of the Hub Genes in UvIM Male Modules, M1 (*Rbf*), M3 (*Skeletor*), M5 (*amos*), and M10 (*Ndf*) and Female Modules F2 (XLOC 13636), F3 (XLOC 1508), F6 (*unc-13*), and F7 (*Ndf*) in *Carabus uenoi*.

Factor	Df	*χ* ^2^	*P*-Value	*χ* ^2^	*P*-Value	*χ* ^2^	*P*-Value	*χ* ^2^	*P*-Value
Male module		M1 (*Rbf*)		M3 (*Skeletor*)		M5 (*amos*)		M10 (*Ndf*)	
Stage	3	36.349	*<0.0001*	29.009	*<0.0001*	86.570	*<0.0001*	34.357	*<0.0001*
Sex	1	3.503	0.0613	0.736	0.3909	43.181	*<0.0001*	1.170	0.2794
Stage*Sex	3	2.678	0.4440	4.589	0.2045	64.104	*<0.0001*	1.480	0.6869
Female module		F2 (XLOC 13636)		F3 (XLOC 1508)		F6 (*unc-13*)		F7 (*Ndf*)	
Stage	3	5.984	0.1124	41.394	*<0.0001*	13.709	*0.0033*	34.357	*<0.0001*
Sex	1	14.814	*0.0001*	7.314	*0.0068*	2.769	0.0961	1.170	0.2794
Stage*Sex	3	1.158	0.7632	24.165	*<0.0001*	13.675	*0.0034*	1.480	0.6869

Italicized *P*-values are < 0.05.

Note.—The top hub genes are indicated in the parentheses. The top hub genes of module M10 and F7 are the same (*Ndf*).

## Discussion

### Interspecific DEGs Showing Sex-Concordant and Sex-Discordant Expression

In a previous study with *C. maiyasanus*, we found that few genes were differentially expressed between the sexes, but that these DEGs were likely related to genital morphogenesis; genes exhibiting sex-specific expression might be involved in sex-specific genital morphology (Nomura et al. 2020). In the present study, we found a small number of DEGs showing large between-sex expression variance (thus, sex-specific or sex-discordant expression), some of which were involved in imaginal disc development, and could therefore be related to genital morphogenesis. These DEGs may be involved in both sex-specific and species-specific morphogenesis of male and female genitalia. In our analysis of interspecific DEGs in each stage of each sex, we found more interspecific DEGs showing sex-concordant expression than sex-discordant expression, except in PL of the IvM comparison. The interspecific DEGs showing sex-concordant expression were relatively abundant at the PpL and PE stages in the UvIM comparison, where sex-concordant expression means that male and female of *C. iwawakianus* and *C. maiyasanus* showed higher or lower gene expression than those of *C. uenoi*. Considering the small sex-related expression variance for the interspecific DEGs in the UvIM comparison, the sex-concordant expression of particular genes may have contributed to the exaggeration of genital size in *C. uenoi*. The sex-concordant gene expression may result in a positive intersexual genetic correlation, which occurs in the genital sizes of beetles (Simmons and Garcia-Gonzalez 2011). In the genital coevolution between the sexes, a positive intersexual genetic correlation in gene expression would facilitate correlated evolution between the sexes.

### Gene Networks Involved in the Interspecific Differences of Genital Morphologies

Because there were many DEGs between species at each stage, we generated gene network modules by WGCNA in each sex and characterized the genes in the modules via GO enrichment analysis to detect modules containing genes that may be related to genital morphology in each sex. We found that two male and three female modules in the IvM comparison, and four male and three female modules in the UvIM comparison, were enriched in GO terms related to genital morphogenesis. In addition to the characterization of modules via GO analysis, we also checked if the modules contained transcription factors or genetic pathways involved in the control of organ morphology and that may be related to genital morphology (Lavine et al. 2015; Nomura et al. 2020). We found that genes associated with genital morphology were involved in two male and one female modules of the IvM comparison, and two male and two female modules in the UvIM comparison.

The genes in the mTOR signaling and hedgehog signaling pathways were found in the male module M1 of the UvIM comparison. Because genes in these pathways are also involved in the control of organ size and morphogenesis (Tumaneng et al. 2012; Villarreal et al. 2015), the genes in the above modules are strong candidates for interspecific differences in genital size and shape in the UvIM comparison. Furthermore, because there were overlaps in the list of hub genes showing top 50 MM between male and female modules in both the IvM and UvIM comparisons, genes in these modules may construct similar gene networks in the male and female genital tissues. These results suggest that genes showing common interactions in male and female genital tissues are strong candidates for interspecific differences in genital size and shape.

### Gene Expression Patterns Related to Species-Specific and Exaggerated Genital Morphology with Coevolution between the Sexes

Our results revealed that interspecific differences in the expression levels of the hub genes in the candidate modules were present at various stages in the modules. In the IvM comparison, interspecific differences of expression levels in the hub genes of male module M5 and female module F6 were found mainly at the PL stage. The same expression profiles were found in male modules M2 and M8 and female modules F2 and F4, although the differences were not significant. A recent micro-CT study that examined the morphogenetic process in the genitalia of *C. iwawakianus* and *C. maiyasanus* during the pupal stage showed that interspecific differences in the genital morphology became visible 4–6 days after pupation for the male copulatory piece, and 6–8 days for the female vaginal appendix (Terada et al. 2021). Therefore, the differential expression of genes contained in male module M5 and female module F6 at the PL stage (4–6 days after pupation) may affect the differences in genital shape between these species. These modules shared several hub genes, although the top hub gene differed for each (*scra* for M5; *Cdk1* for F6), and may represent shared gene networks between the sexes. Interestingly, the expression of the top hub genes at the PL stage differed between the sexes, with higher expression in *C. maiyasanus* males and higher expression in *C. iwawakianus* females. Thus, sex-specific regulatory changes between the species in shared gene networks may have resulted in the species-specific genital morphology in each sex. Because both *scra* and *Cdk1* are involved in mitosis in *Drosophila* (Nurse 1990; Field et al. 2005), the differential expression timing of these genes in the tissues of the copulatory piece or vaginal appendix may result in differences in the frequency of mitosis and produce interspecific differences in genital shapes, such as copulatory piece length and width.

In the UvIM comparison, the hub genes of male module M5 and female module F3 showed interspecific differences in expression levels at the PL stage, as in the IvM comparison, and the expression differed between the sexes in *C. uenoi*. On the other hand, for the hub genes of male modules M1 and M10 and female module F7, there were interspecific differences in expression from the PpL to PL stage, and no distinct differences between sexes of the same species. Because male module M1 contained the mTOR and hedgehog signaling pathways, and male module M10 and female module F7 contained transcription factors involved in organ size control, differences in expression levels of the genes contained in these modules may be involved in the exaggeration of the male and female genital sizes in *C. uenoi*. These results may imply that the development of genital parts progresses earlier in *C. uenoi* than *C. iwawakianus* and *C. maiyasanus*, and is further promoted at the PL stage. The process of genital morphogenesis in *C. uenoi* should be examined by micro-CT to confirm this prediction.

In conclusion, our results from the IvM and UvIM comparisons suggest that gene networks shared by both sexes are involved in the formation of species-specific genitalia, changes in the expression timing profiles of those gene networks between species affect differences in genital size and shape, and the changes in the expression profiles can either be sex-concordant or sex-discordant depending on the traits involved in species divergence. In the IvM comparison, sex-specific (discordant) gene expression is likely to be important, whereas in the UvIM comparison, both sex-concordant and sex-discordant gene expression are likely to be important. Particularly, sex-concordant gene expression may be important for the coevolution of the exaggerated male and female genitalia in *C. uenoi*.

In *Ohomopterus*, matching between the copulatory piece and vaginal appendix is subject to sex-concordant natural selection to avoid genital injury (Sota and Kubota 1998), although selection for a longer male genital part in sperm competition (Takami and Sota 2007) may cause sexual conflict over the lengths of male and female genital parts (Takami et al. 2018). In addition, stronger sex-concordant selection can arise from the need to avoid maladaptive interspecific hybridization, and this may have been the major cause of the exaggeration of genital size in *C. uenoi*; this species is sympatric with *C. iwawakianus*, but no hybridization is observed probably because of the excessive difference in genital size, implying the past occurrence of reinforcing selection (Sota and Kubota 1998; Okuzaki and Sota 2014). The coevolution toward exaggerated genitalia in *C. uenoi* may have been facilitated by the evolution of the gene network involved in genital morphogenesis, which is shared between the sexes showing sex-concordant expression profiles. Thus, our study illuminates the possible genetic mechanism of concerted coevolution toward exaggeration between male and female genital morphology.

## Materials and Methods

### Sample Preparation

We collected adult *Carabus* (*Ohomopterus*) *maiyasanus* at Mt. Uryu, Kyoto, and both *C*. *iwawakianus* and *C*. *uenoi* at Mt. Kongo, Osaka in May–June 2016 and reared them at 20 °C under long-day conditions (light:dark [LD], 16:8 h) to obtain larvae and pupae for the transcriptome study. Parental females were reared individually in 12-cm-diameter, 9.5-cm-deep plastic cups with a 4-cm-deep soil layer and were fed minced beef. Eggs deposited in the soil were collected and incubated at 20 °C and LD 16:8 h. After hatching, the larvae were reared individually in 9-cm-diameter, 4-cm-deep plastic cups and fed megascolecid earthworms. When the third (last) instar larvae were fully grown, they were transferred to 6.5-cm-diameter, 7.5-cm-deep plastic cups with 6-cm-deep soil to allow them to pupate in the soil; they made cavities in the soil and became prepupae and then pupae. The third instar larvae pupate ∼7 days after burrowing into the soil and emerge ∼10 days after pupation. In male pupae, the apical part of the aedeagus protrudes from the tip of the abdomen, and the entire aedeagus becomes visible as the pupal period progresses. Thus, pupal sex is easily judged by the presence or absence of an aedeagus tip.

We fixed third instar larvae and pupae in RNAlater solution (Invitrogen, Carlsbad, CA, USA) at the following four stages: 1) early prepupa (PpE), third instar larvae 1–3 days after burrowing into the soil; 2) late prepupa (PpL), third instar larvae 4–6 days after burrowing into the soil (2–3 days before pupation); 3) early pupa (PE), pupae 1–3 days after pupation; and 4) late pupa (PL), pupae 4–6 days after pupation. For each stage, we obtained at least six samples, so that three samples were available for each sex after sex determination using a molecular marker (see below). Samples fixed in RNAlater were stored at −80 °C until RNA extraction.

### RNA Extraction, Sex Determination, and RNA Sequencing

As in our previous study (Nomura et al. 2020), total RNA was extracted from the tissue of abdominal segments A9–11 in larvae and the genital parts in pupae (fig. 1*C*). For RNA extraction, we used the RNeasy Mini Kit (QIAGEN, Hilden, Germany), following the manufacturer’s protocol. We treated the extract with DNase I for 15 min at room temperature to remove genomic DNA.

We determined the sex of larval samples using a molecular method based on the length difference of the PCR products of the *dsx* gene due to sex-specific isoforms (Nomura et al. 2020) because morphological identification cannot be performed on larvae. The total number of samples after the sex determination was 72 (i.e., 3 samples × 2 sexes × 4 stages × 3 species). Sequence libraries were constructed and sequenced by the Beijing Genomic Institute (BGI) and Novogene using the sequencing platforms Illumina HiSeq 2500 (100 bp, paired-end, 30 M reads per sample) and Illumina HiSeq 4000 (150 bp, paired-end, 30 M reads per sample), respectively. All raw read data have been deposited in the DNA Data Bank of the Japan Sequence Reach Archive (BioProject PRJDB5403; supplementary table S1, Supplementary Material online).

### Sequence Data Quality Control, Assembly, and Read Counts

The quality of the sequence reads was evaluated using FastQC v. 0.11.5 (Andrews 2010). Because read lengths differed between the sequencing platforms, 50 bp were trimmed from the 150-bp reads at the 3′-end using PRINSEQ v. 0.20.4 (Schmieder and Edwards 2011). The 100-bp reads were mapped to the reference genome sequence of *C. uenoi* (Fujisawa et al. 2019) using the paired-end option in Bowtie 2 v. 2.2.9 (Langmead and Salzberg 2012) and Top Hat 2 v. 2.1.1 (Kim et al. 2013). The *C*. *uenoi* genome sequence was the only draft genome available among the three species for read count normalization. We obtained similar mapping rates for all samples (supplementary table S1, Supplementary Material online). To account for the effect of species-specific single-nucleotide polymorphisms (SNPs), we masked SNPs between species found in the *C*. *uenoi* genome with N. We assembled mapped reads in protein-coding regions using cuffmerge v. 2.2.1 in the cufflinks package (Trapnell et al. 2012), and the protein-coding regions were matched to *Drosophila melanogaster* RefSeq proteins using blastx (E-value < 1e^−5^). We obtained read count data using featureCounts v. 1.5.1 (Liao et al. 2014) to estimate gene expression levels.

### Gene Expression Variance Analysis

We evaluated the contributions of potential variables, developmental stage, species, and sex to the obtained gene expression variance using a linear model implemented in the variancePartition v. 1.18.3 package in R (Hoffman and Schadt 2016). All variables were modeled as random effects because these variables are categorical. The linear model is as follows:

Gene expression ∼ (1|Stages)+(1|Species)+(1|Sex).

We also performed PCA for the 72 samples to summarize the variation in gene expression patterns among samples (supplementary fig. S2, Supplementary Material online). Scores obtained for PC1 and PC2 were tested using a generalized linear model (GLM) to investigate differences by species, sex, or stage (supplementary table S2, Supplementary Material online). Before the gene expression variation analysis, we normalized read counts using the TCC v. 1.14.0 (Sun et al. 2013) package in R and converted them to *Z*-scores.

### DEGs between Species and Co-Expression Network Analysis for Module Construction

We performed pairwise differential gene expression analysis between *C*. *iwawakianus* and *C*. *maiyasanus* (the IvM comparison) and between *C*. *uenoi* and *C*. *iwawakianus* or *C*. *maiyasanus* (the UvIM comparison) for each developmental stage and sex using the DESeq2 v. 1.14.1 (Love et al. 2014) package in R (R Core Team 2000); FDR < 0.05 was used to define DEGs. In the UvIM comparison, we obtained the DEGs that were differentially expressed in both *C*. *iwawakianus* versus *C*. *uenoi* and *C*. *maiyasanus* versus *C*. *uenoi* at the same stage and sex. Thus, we obtained DEGs between species that showed different expression levels in either sex. For DEGs with >5% variance explained by sex, GO enrichment analysis were performed using Metascape (Zhou et al. 2019).

We extracted gene co-expression networks (modules), which are clusters of genes with similar expression patterns, using the WGCNA v. 1.68 package in R (Langfelder and Horvath 2008) for the DEGs in the IvM and UvIM comparisons. WGCNA is a useful tool for performing adaptation and speciation studies (Frisch et al. 2020; Morgan et al. 2020). Because male and female genitalia are not strictly homologous and may be formed as a result of sex-specific interactions among genes, we performed WGCNA in each sex for 3,895 and 7,031 DEGs in the IvM and UvIM comparisons, respectively. First, we drew the clustering dendrogram from the Euclidian distance of the expression levels for all samples and confirmed no outliers (supplementary figs. S3 and S4, Supplementary Material online). We selected 9 in the IvM comparison and 14 in UvIM comparison of both sexes as the optimal soft thresholding powers for module construction (supplementary figs. S5 and S6, Supplementary Material online). For each module obtained, GO enrichment analysis and Kyoto Encyclopedia of Genes and Genome (KEGG) pathway analysis were performed using Metascape, and the terms with FDR < 0.01 were identified as the functions of the genes included in the module.

### Hub Genes in the Modules

We assumed that the hub genes of the modules we selected for further analysis based on their GO analysis results played a central role within the module gene networks, and thus were identified as candidate genes involved in differences in species-specific genital morphology. For each module, the top 50 genes with the highest MM values calculated from the co-expression network analysis were classed as hub genes. The MM of each gene was calculated with the correlation of the module eigengene (summary profile of the module) and the gene expression profile (Langfelder and Horvath 2008). To estimate the average expression profiles of genes in the network, we obtained expression profiles across the four developmental stages using the *Z*-score of the gene with the highest MM (i.e., top hub gene) as the representative of all genes within a module. We tested differences in expression among species for each stage using Tukey’s honest significant difference test. The effects of developmental stage, sex, and species on hub gene expression levels were examined using the GLM. In the IvM comparison, we examined the effects of stage, sex, and species on the expression levels of the hub genes in *C. iwawakianus* and *C. maiyasanus*, and in the UvIM comparison, we examined the effects of stage and sex on the expression levels in *C. uenoi*.

## Supplementary Material

msab122_Supplementary_Data
