# Erratum to: Role of Sex-Concordant Gene Expression in the Coevolution of Exaggerated Male and Female Genitalia in a Beetle Group

**DOI:** 10.1093/molbev/msab167

**Published:** 2021-08-02

**Authors:** Shota Nomura, Tomochika Fujisawa, Teiji Sota

*Molecular Biology and Evolution*, msab122, https://doi.org/10.1093/molbev/msab122

In the originally published version of this manuscript, there was an error in Table 3. The second line from the bottom of the table read “UvIM Male Module M1”. It should have read “UvIM Male Module M5”. This error has been corrected.

