# Supplementary material for: Role of Sex-Concordant Gene Expression in the Coevolution of Exaggerated Male and Female Genitalia in a Beetle Group
Source: Mol Biol Evol. 2021 Apr 27;38(9):3593–605. doi: 10.1093/molbev/msab122 (PMC8382896; doi:10.1093/molbev/msab122)
Supplement: msab122_Supplementary_Data [file msab122_supplementary_data.zip › Covering letter210323.docx]

Dear Editors, Molecular Biology and Evolution,

Thank you very much for kindly reviewing our manuscript entitled “The role of sex-concordant gene expression in the coevolution of exaggerated male and female genitalia in a beetle group”. We appreciated comments and suggestions by reviewers and you and have revised our manuscript carefully considering all of them. We performed the gene expression network analysis separately for each sex and have abandoned use of PPI for determining hub genes. Thus, we have reanalyzed our data and revised the manuscript extensively. Changes in the text are indicated in blue letters. Our responses to editors’ and reviewers’ comments are described separately.

No part of this manuscript has been published or is under consideration for publication in another journal or book. All persons entitled to authorship have been so named, and all authors have approved the final version of the manuscript.

We would much appreciate your kind consideration of our revised manuscript for publication in Molecular Biology and Evolution.

Yours sincerely,

Teiji Sota, corresponding author

Department of Zoology, Graduate School of Science

Kyoto University

Kitashirakawa-oiwake-cho, Sakyo-ku, Kyoto 606-8502, Japan

Email: sota@terra.zool.kyoto-u.ac.jp

Tel. +81-75-753-4078
