# Supplementary material for: Role of Sex-Concordant Gene Expression in the Coevolution of Exaggerated Male and Female Genitalia in a Beetle Group
Source: Mol Biol Evol. 2021 Apr 27;38(9):3593–605. doi: 10.1093/molbev/msab122 (PMC8382896; doi:10.1093/molbev/msab122)
Supplement: msab122_Supplementary_Data [file msab122_supplementary_data.zip › Supple_bind_210421.pdf]

Supplementary materials for:

Role of sex-concordant gene expression in the coevolution of exaggerated male and female genitalia in a beetle group

Shota Nomura[1], Tomochika Fujisawa[1,2] and Teiji Sota[1]

[1] Department of Zoology, Graduate School of Science, Kyoto University, Sakyo, Kyoto, 606-8502, Japan.

[2] The Center for Data Science Education and Research, Shiga University, Hikone, Shiga, 522-8522, Japan.

Contents

Fig. S1. Percentages of the variance in gene expression explained by stage, species, and sex differences for all 18,839 genes in the three species.

Fig. S2. The relationships between the first and second principal component scores (PC1 vs. PC2) with read counts data from individual samples.

Fig. S3. The sample clustering tree based on the expression level of DEGs in the IvM comparison.

Fig. S4. The sample clustering tree based on the expression level of DEGs in the UvIM comparison.

Fig. S5. The soft thresholding power of co-expression network analysis in the IvM comparison.

Fig. S6. The soft thresholding power of co-expression network analysis in the UvIM comparison.

Table S1. Details of RNA-seq samples and read data for *Carabus iwawakianus*, *C. maiyasanus* and *C. uenoi*.

Table S2. Results of the generalized linear model (GLM) analysis of principal component scores.

Table S3. Genes with the "imaginal disc development" among genes that showed large expression variation between the sexes in the IvM comparison.

Table S4. Genes with the "imaginal disc development" among genes that showed large expression variation between the sexes in the UvIM comparison.

Table S5. Numbers of genes in each module, annotated and are used for GO analysis in the IvM.

Table S6. Number of genes in each module, annotated and are used for GO analysis in

the UvIM.

Table S7. The top 50 genes among the module memberships (MM) calculated from co-expression network analysis in modules of the IvM comparison.

Table S8. The top 50 genes among the module memberships (MM) calculated from co-expression network analysis in modules of the UvIM comparison.

Fig. S1. Percentages of the variance in gene expression explained by stage, species, and sex differences for all 18,839 genes in the three species.

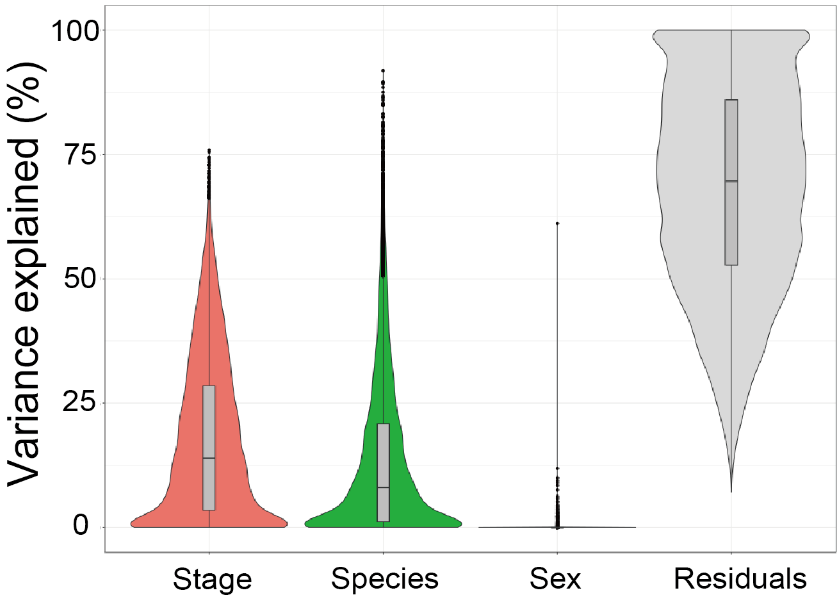

Fig. S2. The relationships between the first and second principal component scores (PC1 vs. PC2) resulting from the principal component analysis with read counts data from individual samples. The proportion of variance explained by each principle component is given in parentheses. Open square, male early prepupa (PpE); filled square, female PpE; open circle, male late prepupa (PpL); filled circle, female PpL; open triangle, male early pupa (PE); filled triangle, female PE; open diamond, male late pupa (PL); filled diamond, female PL; green, *C. iwawakianus*; red, *C. maiyasanus*; blue, *C. uenoi*.

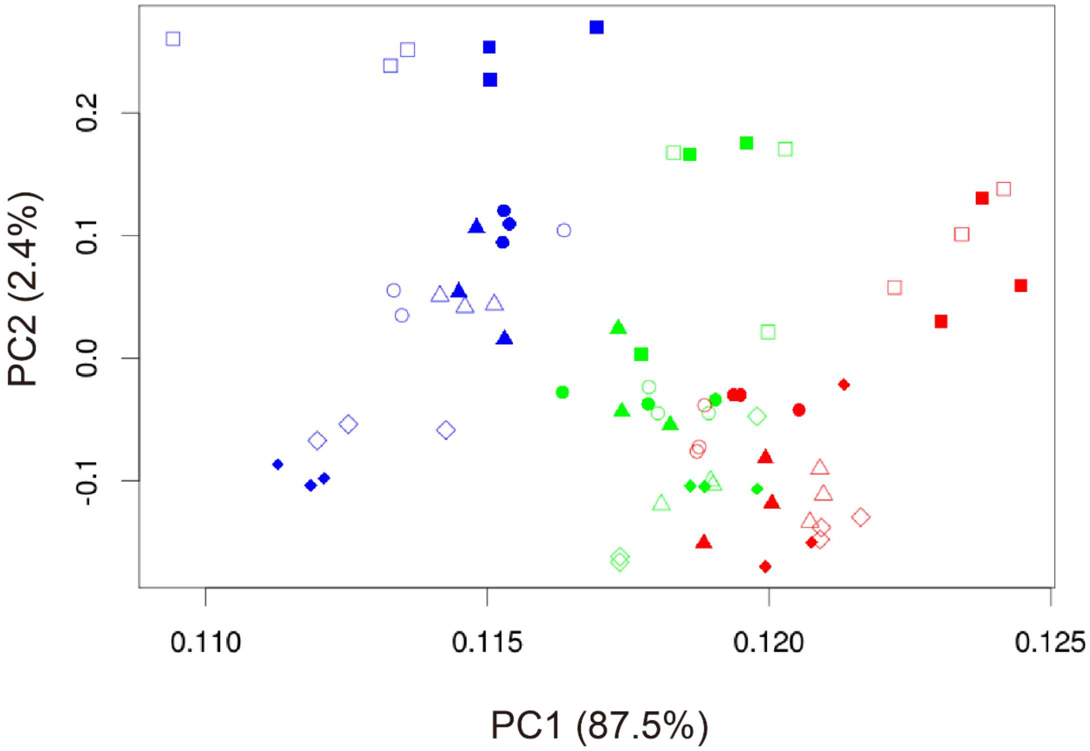

Fig. S3. The sample clustering tree based on the expression level of DEGs in male (*A*) and female (*B*) of the IvM comparison (n = 3,895).

A male

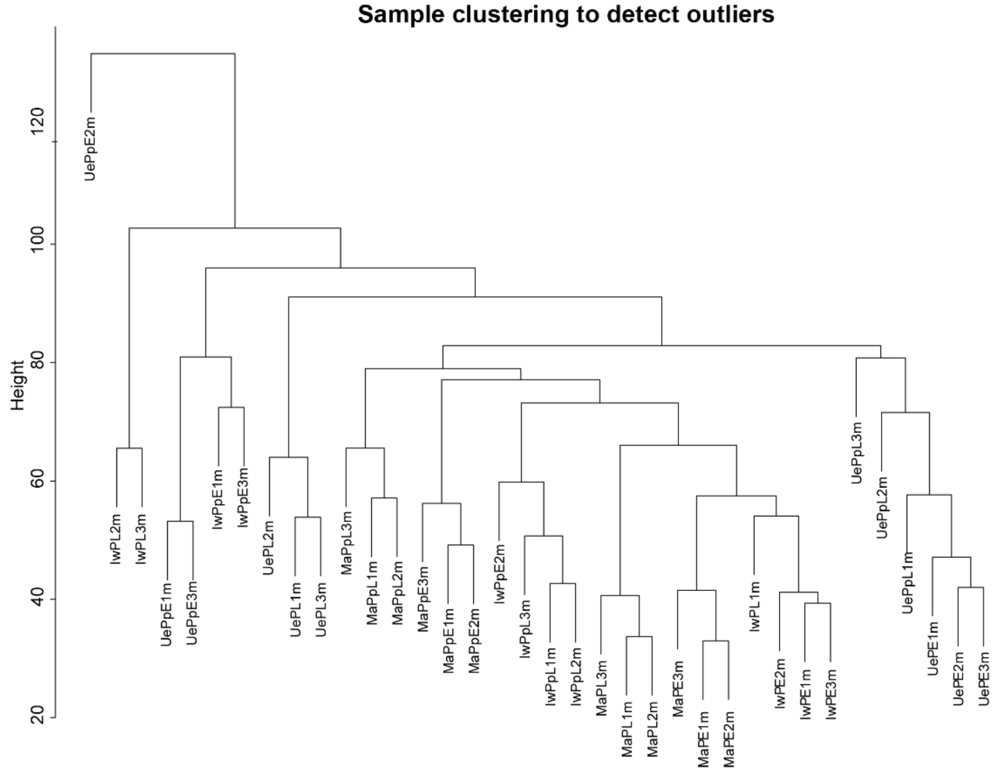

B female

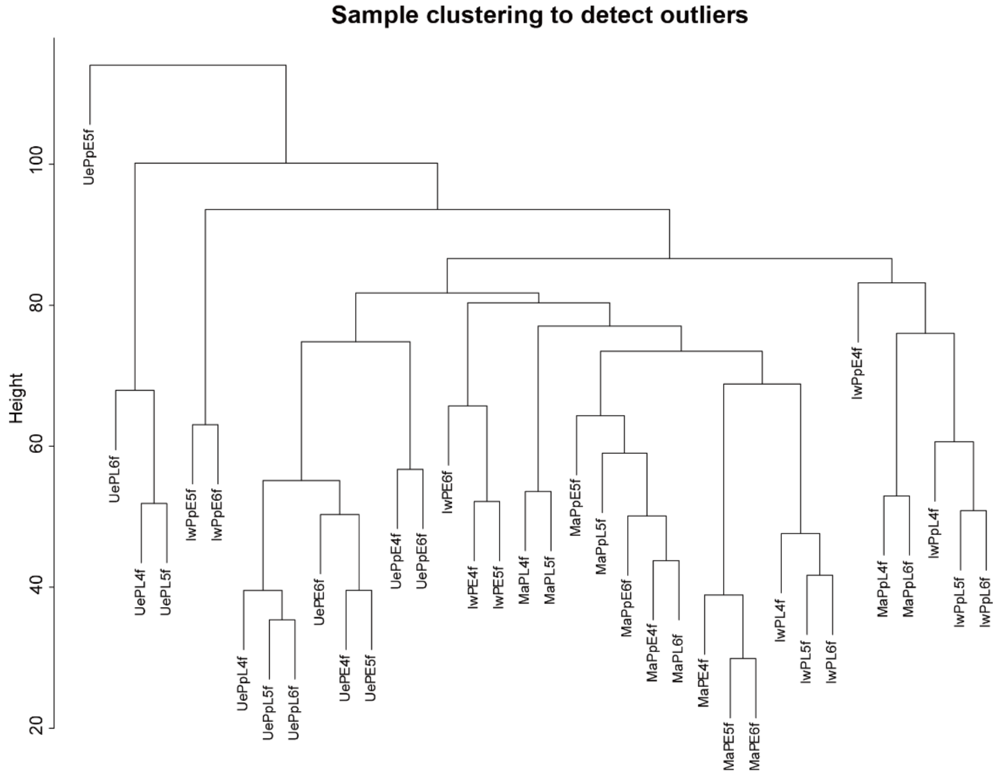

Fig. S4. The sample clustering tree based on the expression level of DEGs in male (*A*) and female (*B*) of the UvIM comparison (*n* = 7,031).

A male

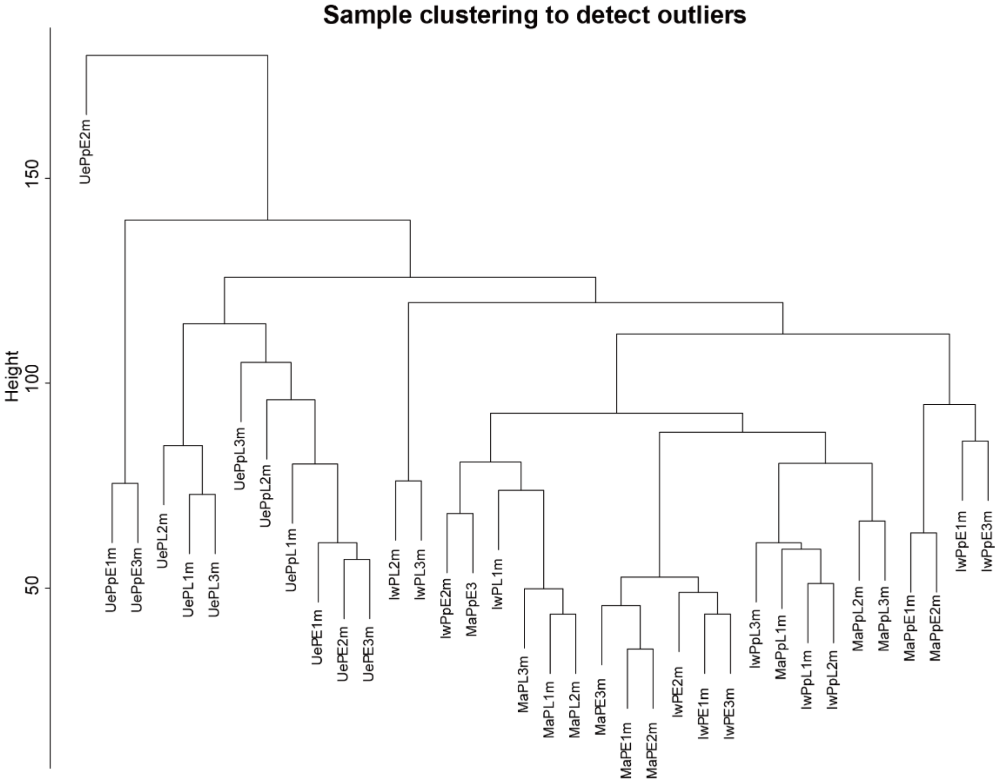

B female

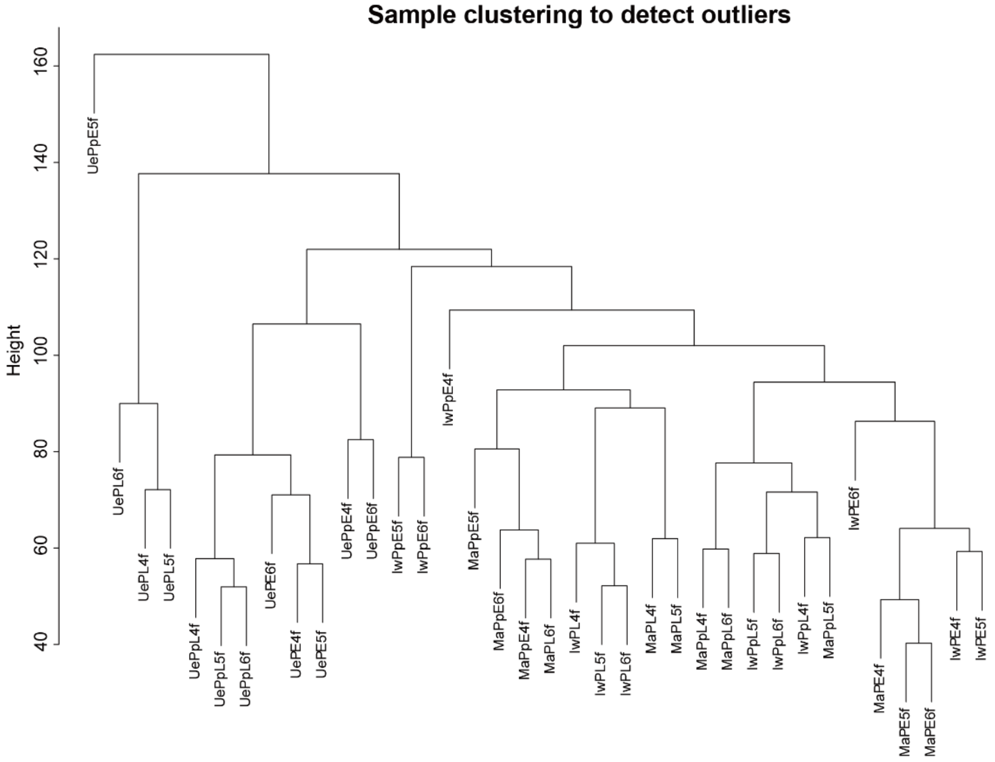

Fig. S5. Selection of the soft thresholding power of co-expression network analysis in male (*A*) and female (*B*) of the IvM comparison based on the criterion of approximate scale-free topology.

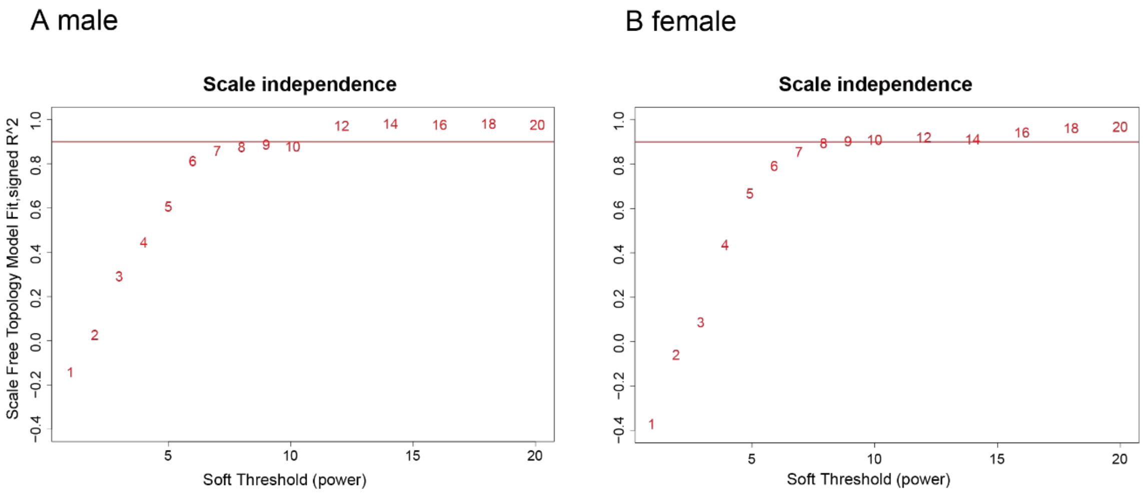

Fig. S6. Selection of the soft thresholding power of co-expression network analysis in male (*A*) and female (*B*) of the UvIM comparison based on the criterion of approximate scale-free topology.

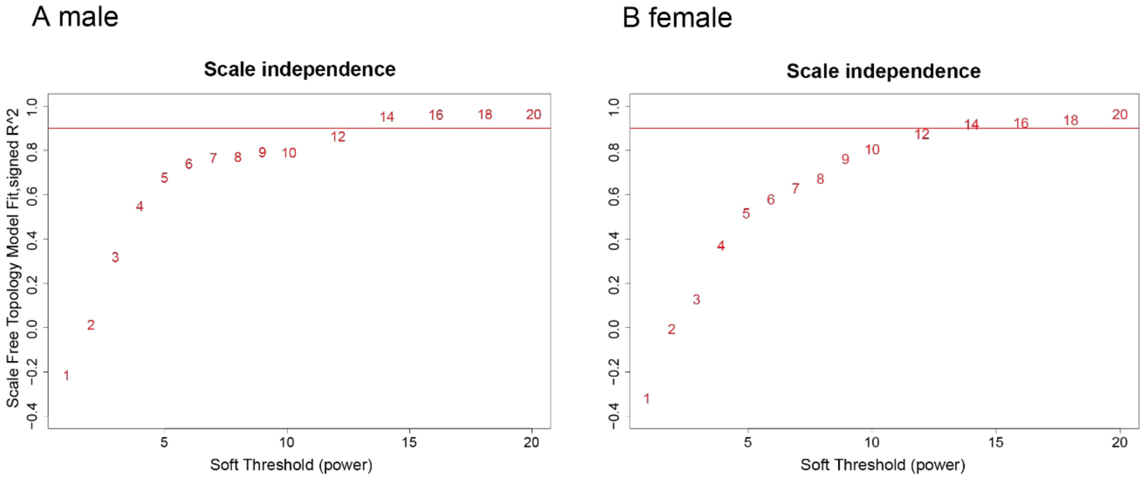

Table S1 Details of RNA-seq samples and read data for *Carabus iwawakianus, C. maiyasanus* and *C. uenoi* third instar larvae (prepupae) and pupae.

					Raw read metrics		Mapped read metrics		
Sample	Stage1	Species	Sex	Sequence platform	Raw read pairs	Average phred score	Mapped read	Mapping rate(%)	DDBJ Biosample accession number2
IwPpE1m	PpE	*C. iwawakianus*	male	HiSeq 4000	13469273	40	9178705	34.1	SAMD00245659
IwPpE2m	PpE	*C. iwawakianus*	male	HiSeq 4000	14466277	40	11325889	39.1	SAMD00245660
IwPpE3m	PpE	*C. iwawakianus*	male	HiSeq 4000	12982504	40	9633411	37.1	SAMD00245661
IwPpE4f	PpE	*C. iwawakianus*	female	HiSeq 4000	17322827	40	11659563	33.7	SAMD00245662
IwPpE5f	PpE	*C. iwawakianus*	female	HiSeq 4000	13866523	40	10273125	37	SAMD00245663
IwPpE6f	PpE	*C. iwawakianus*	female	HiSeq 4000	15554788	40	11760645	37.8	SAMD00245664
IwPpL1m	PpL	*C. iwawakianus*	male	HiSeq 2500	11724235	38	9663088	41.2	SAMD00119564
IwPpL2m	PpL	*C. iwawakianus*	male	HiSeq 2500	11849527	38	9853296	41.6	SAMD00119565
IwPpL3m	PpL	*C. iwawakianus*	male	HiSeq 2500	11349566	38	8674320	38.2	SAMD00119566
IwPpL4f	PpL	*C. iwawakianus*	female	HiSeq 2500	12937201	38	10684117	41.3	SAMD00119561
IwPpL5f	PpL	*C. iwawakianus*	female	HiSeq 2500	11788908	38	9715069	41.2	SAMD00119562
IwPpL6f	PpL	*C. iwawakianus*	female	HiSeq 2500	11816899	38	9630729	40.7	SAMD00119563
IwPE1m	PE	*C. iwawakianus*	male	HiSeq 2500	11661634	38	9512291	40.8	SAMD00119570
IwPE2m	PE	*C. iwawakianus*	male	HiSeq 2500	14129203	38	11601689	41.1	SAMD00119571
IwPE3m	PE	*C. iwawakianus*	male	HiSeq 2500	12350533	38	10016674	40.6	SAMD00119572
IwPE4f	PE	*C. iwawakianus*	female	HiSeq 2500	14029442	38	10804902	38.5	SAMD00119567
IwPE5f	PE	*C. iwawakianus*	female	HiSeq 2500	13534913	38	10798459	39.9	SAMD00119568
IwPE6f	PE	*C. iwawakianus*	female	HiSeq 2500	13126142	38	9558542	36.4	SAMD00119569
IwPL1m	PL	*C. iwawakianus*	male	HiSeq 4000	12463422	40	8881115	35.6	SAMD00245665
IwPL2m	PL	*C. iwawakianus*	male	HiSeq 4000	13775439	40	10327958	37.5	SAMD00245666
IwPL3m	PL	*C. iwawakianus*	male	HiSeq 4000	12491428	40	8823448	35.3	SAMD00245667
IwPL4f	PL	*C. iwawakianus*	female	HiSeq 4000	12019607	40	9397950	39.1	SAMD00245668
IwPL5f	PL	*C. iwawakianus*	female	HiSeq 4000	12296213	40	9642521	39.2	SAMD00245669
IwPL6f	PL	*C. iwawakianus*	female	HiSeq 4000	10704130	40	8402654	39.2	SAMD00245670
MaPpE1m	PpE	*C. maiyasanus*	male	HiSeq 4000	13008803	40	8544891	32.8	SAMD00165705

MaPpE2m	PpE	*C. maiyasanus*	male	HiSeq 4000	12438000	40	8403529	33.8	SAMD00165706
MaPpE3m	PpE	*C. maiyasanus*	male	HiSeq 4000	13037724	40	8606288	33.0	SAMD00165707
MaPpE4f	PpE	*C. maiyasanus*	female	HiSeq 4000	11083533	40	7880811	35.6	SAMD00165708
MaPpE5f	PpE	*C. maiyasanus*	female	HiSeq 4000	12728730	40	8527174	33.5	SAMD00165709
MaPpE6f	PpE	*C. maiyasanus*	female	HiSeq 4000	10407638	40	7184421	34.5	SAMD00165710
MaPpL1m	PpL	*C. maiyasanus*	male	HiSeq 2500	13519968	38	10764149	39.8	SAMD00119576
MaPpL2m	PpL	*C. maiyasanus*	male	HiSeq 2500	12404308	38	8863606	35.7	SAMD00119577
MaPpL3m	PpL	*C. maiyasanus*	male	HiSeq 2500	12867043	38	9461606	36.8	SAMD00119578
MaPpL4f	PpL	*C. maiyasanus*	female	HiSeq 2500	11401683	38	8380196	36.7	SAMD00119573
MaPpL5f	PpL	*C. maiyasanus*	female	HiSeq 2500	13602549	38	10690791	39.3	SAMD00119574
MaPpL6f	PpL	*C. maiyasanus*	female	HiSeq 2500	11927164	38	9061264	38.0	SAMD00119575
MaPE1m	PE	*C. maiyasanus*	male	HiSeq 2500	12039814	38	9291447	38.6	SAMD00119582
MaPE2m	PE	*C. maiyasanus*	male	HiSeq 2500	13104051	38	10128688	38.6	SAMD00119583
MaPE3m	PE	*C. maiyasanus*	male	HiSeq 2500	12920295	38	9995793	38.7	SAMD00119584
MaPE4f	PE	*C. maiyasanus*	female	HiSeq 2500	14151603	38	10692109	37.8	SAMD00119579
MaPE5f	PE	*C. maiyasanus*	female	HiSeq 2500	13632467	38	10947500	40.2	SAMD00119580
MaPE6f	PE	*C. maiyasanus*	female	HiSeq 2500	15037197	38	12186343	40.5	SAMD00119581
MaPL1m	PL	*C. maiyasanus*	male	HiSeq 4000	13507984	40	10282267	38.1	SAMD00165711
MaPL2m	PL	*C. maiyasanus*	male	HiSeq 4000	13178545	40	9630120	36.5	SAMD00165712
MaPL3m	PL	*C. maiyasanus*	male	HiSeq 4000	12041532	40	9153066	38.0	SAMD00165713
MaPL4f	PL	*C. maiyasanus*	female	HiSeq 4000	12259409	40	9494932	38.7	SAMD00165714
MaPL5f	PL	*C. maiyasanus*	female	HiSeq 4000	12883100	40	9740743	37.8	SAMD00165715
MaPL6f	PL	*C. maiyasanus*	female	HiSeq 4000	13512773	40	9200397	34.0	SAMD00165716
UePpE1m	PpE	*C. uenoi*	male	HiSeq 4000	13104049	40	10942173	41.8	SAMD00245671
UePpE2m	PpE	*C. uenoi*	male	HiSeq 4000	14770015	39	9809256	33.2	SAMD00245672
UePpE3m	PpE	*C. uenoi*	male	HiSeq 4000	12801833	40	10454359	40.8	SAMD00245673
UePpE4f	PpE	*C. uenoi*	female	HiSeq 4000	13349518	40	11787316	44.1	SAMD00245674
UePpE5f	PpE	*C. uenoi*	female	HiSeq 4000	13708352	40	9105627	33.2	SAMD00245675
UePpE6f	PpE	*C. uenoi*	female	HiSeq 4000	13971344	40	12248759	43.8	SAMD00245676
UePpL1m	PpL	*C. uenoi*	male	HiSeq 4000	10041342	40	8350297	41.6	SAMD00245677

UePpL2m	PpL	*C. uenoi*	male	HiSeq 4000	13781452	40	11101946	40.3	SAMD00245678
UePpL3m	PpL	*C. uenoi*	male	HiSeq 4000	12778583	40	10594935	41.5	SAMD00245679
UePpL4f	PpL	*C. uenoi*	female	HiSeq 4000	14954018	40	12873152	43.0	SAMD00245680
UePpL5f	PpL	*C. uenoi*	female	HiSeq 4000	14438218	40	12359675	42.8	SAMD00245681
UePpL6f	PpL	*C. uenoi*	female	HiSeq 4000	14115511	40	11751535	41.6	SAMD00245682
UePE1m	PE	*C. uenoi*	male	HiSeq 4000	13784150	40	10493127	38.1	SAMD00245683
UePE2m	PE	*C. uenoi*	male	HiSeq 4000	11580292	40	9504924	41.0	SAMD00245684
UePE3m	PE	*C. uenoi*	male	HiSeq 4000	12645994	40	10439575	41.3	SAMD00245685
UePE4f	PE	*C. uenoi*	female	HiSeq 4000	14630293	40	13043263	44.6	SAMD00245686
UePE5f	PE	*C. uenoi*	female	HiSeq 4000	13164492	40	11274996	42.8	SAMD00245687
UePE6f	PE	*C. uenoi*	female	HiSeq 4000	14090843	40	12204356	43.3	SAMD00245688
UePL1m	PL	*C. uenoi*	male	HiSeq 4000	12713099	40	11296604	44.4	SAMD00245689
UePL2m	PL	*C. uenoi*	male	HiSeq 4000	11675185	40	9445973	40.5	SAMD00245690
UePL3m	PL	*C. uenoi*	male	HiSeq 4000	12567896	40	11204487	44.6	SAMD00245691
UePL4f	PL	*C. uenoi*	female	HiSeq 4000	14502616	40	13983654	48.2	SAMD00245692
UePL5f	PL	*C. uenoi*	female	HiSeq 4000	12759439	39	12385783	48.5	SAMD00245693
UePL6f	PL	*C. uenoi*	female	HiSeq 4000	12461912	40	11592372	46.5	SAMD00245694

1) PpE, third instar early prepupa; PpL, third instar late prepupa; PE, early pupa; PL, late pupa.

2) BioProject ID: PRJDB5403.

Table S2 Results of the generalized linear model (GLM) analysis of principal component scores in *C. iwawakianus*, *C. maiyasanus* and *C. uenoi*.

	Estimate	Std.	*t*	*P*
PC1				
Intercept	0.11957	0.00045	265.639	< 2E-16
Species (*C. maiyasanus*)	0.00252	0.00042	6.044	8.15E-08
Species (*C. uenoi*)	-0.00451	0.00042	-10.833	3.35E-16
Stage (PpL)	-0.00145	0.00048	-3.006	0.00376
Stage (PE)	-0.00111	0.00048	-2.312	0.02394
Stage (PL)	-0.00154	0.00048	-3.200	0.00213
Sex (Male)	-0.00015	0.00034	-0.447	0.65623
PC2				
Intercept	0.13165	0.01390	9.470	7.42E-14
Species (*C. maiyasanus*)	-0.02577	0.01287	-2.002	0.0494
Species (*C. uenoi*)	0.10240	0.01287	7.956	3.49E-11
Stage (PpL)	-0.15014	0.01486	-10.102	5.93E-15
Stage (PE)	-0.19391	0.01486	-13.048	< 2E-16
Stage (PL)	-0.25745	0.01486	-17.323	< 2E-16
Sex (Male)	-0.01209	0.01051	-1.151	0.2541

Table S3 Genes associated with the GO term “imaginal disc development” among 283 genes that showed large expression variation between the sexes (expression variance >5%) in the IvM comparison. In the GO analysis, the term “imaginal disc development” was not significantly enriched (log [FDR-*P*] = -1.784).

ID	Gene name	Stage	Species	Sex	Residuals
XLOC_001758	thickveins	0.169	0.209	0.207	0.415
XLOC_003017	drumstick	0.318	0.108	0.094	0.480
XLOC_003719	spalt-related	0.476	0.053	0.094	0.378
XLOC_003800	dystrophin	0.364	0.134	0.081	0.421
XLOC_003811	spalt major	0.368	0.080	0.127	0.424
XLOC_004679	no ocelli	0.144	0.387	0.056	0.413
XLOC_005495	engrailed	0.245	0.072	0.395	0.288
XLOC_005641	tolkin	0.412	0.003	0.053	0.532
XLOC_007079	suppressor of deltex	0.000	0.605	0.063	0.332
XLOC_008623	dachshund	0.258	0.026	0.245	0.470
XLOC_008691	eyegone	0.260	0.008	0.143	0.589
XLOC_010248	protein kinase, cAMP-dependent, catalytic subunit 1	0.194	0.172	0.087	0.547
XLOC_011083	silver	0.363	0.194	0.070	0.373
XLOC_011146	vrille	0.371	0.000	0.063	0.566
XLOC_011201	rotund	0.385	0.189	0.085	0.341
XLOC_011358	B4	0.356	0.053	0.176	0.415
XLOC_011655	transcription factor AP-2	0.409	0.037	0.080	0.474
XLOC_012809	LIM homeobox 1	0.180	0.021	0.411	0.387
XLOC_017158	nab	0.023	0.099	0.219	0.659
XLOC_017278	single-minded	0.141	0.019	0.114	0.727

Table S4 Genes associated with the GO term “imaginal disc development” among 541 genes that showed large expression variation between the sexes (expression variance >5%) in the UvIM comparison. In the GO analysis, the term “imaginal disc development” was significantly enriched (log [FDR-*P*] = -10.161).

ID	Gene name	Stage	Species	Sex	Residuals
XLOC_000141	intersex	0.1194283	0.5301217	0.1046602	0.2457898
XLOC_000584	cubitus interruptus	0.3330982	0.0980193	0.0738007	0.4950818
XLOC_000594	apterous	0.1960839	0.1593565	0.1102331	0.5343265
XLOC_000981	rhea	0.0503392	0.5827024	0.0540412	0.3129172
XLOC_001745	downstream of receptor kinase	0.4074058	0.0876528	0.0550258	0.4499156
XLOC_001758	thickveins	0.1688448	0.2085246	0.2073584	0.4152722
XLOC_001875	axin	0.1843974	0.5279352	0.0632747	0.2243928
XLOC_001914	neuroglian	0.0548935	0.262447	0.1091059	0.5735537
XLOC_002053	steppke	0	0.2882895	0.0513621	0.6603485
XLOC_003003	nuclear fallout	0.0634163	0.066082	0.0507387	0.819763
XLOC_003133	myosin 61F	0.0645144	0.1317453	0.0612147	0.7425257
XLOC_003158	alhambra	0.2157218	0.1428809	0.0536284	0.5877688
XLOC_003238	Glutamate-cysteine ligase catalytic subunit	0.0989618	0.0059439	0.0576475	0.8374467
XLOC_003719	spalt-related	0.4756415	0.0527645	0.0938547	0.3777394
XLOC_003800	dystrophin	0.3640971	0.1343152	0.0805073	0.4210804
XLOC_003811	spalt major	0.3680118	0.0802742	0.1272176	0.4244964
XLOC_004679	no ocelli	0.1441821	0.3867612	0.0557825	0.4132742
XLOC_004728	abdominal A	0.2953115	0.0089902	0.2232581	0.4724402
XLOC_004749	carrier of wingless	0.2364339	0.0688494	0.113373	0.5813437
XLOC_005382	brother of ihog	0.3310488	0.1334784	0.220337	0.3151358
XLOC_005422	dumpy	0.4416209	0	0.0861556	0.4722235
XLOC_005489	invected	0.2237035	0.0350951	0.4041877	0.3370137
XLOC_005495	engrailed	0.244928	0.0724308	0.3948196	0.2878216
XLOC_005641	tolkin	0.4124873	0.0029283	0.0528476	0.5317368
XLOC_006264	trithorax	0	0.5131465	0.0833822	0.4034713
XLOC_006662	midline	0.1564156	0.0160374	0.3818717	0.4456753
XLOC_006821	odd paired	0.1748399	0	0.0643656	0.7607944
XLOC_006938	aristaless	0.3748249	0.0776823	0.2197827	0.3277101
XLOC_007079	suppressor of deltex	0	0.6054668	0.0625386	0.3319946
XLOC_007363	ladybird early	0.2944658	0.102676	0.1445475	0.4583107
XLOC_008691	eyegone	0.2601024	0.0076982	0.142886	0.5893135
XLOC_009018	Puratrophin-1-like	0.2370016	0.0865273	0.1305455	0.5459257
XLOC_009045	bric a brac 2	0.1677061	0.0117473	0.0811663	0.7393804
XLOC_009259	tailup	0.3548861	0.1369115	0.058401	0.4498014
XLOC_009492	doublesex	0.4040925	0.0576398	0.2410886	0.2971791
XLOC_009543	prickle	0.5665105	0.0564277	0.0539983	0.3230635
XLOC_010719	capicua	0.1819948	0.0655994	0.0796458	0.6727601
XLOC_011083	silver	0.3633322	0.1938249	0.0700742	0.3727686
XLOC_011091	cut	0.4729242	0.09056	0.1023255	0.3341903
XLOC_011146	vrille	0.370992	0	0.0633596	0.5656484
XLOC_011201	rotund	0.3853052	0.1891332	0.0845957	0.340966
XLOC_011358	B4	0.3563732	0.0526319	0.1764077	0.4145872
XLOC_013324	MYPT-75D	0.0998144	0.1713014	0.0615548	0.6673293
XLOC_013736	star	0.4307687	0	0.0823915	0.4868398
XLOC_015089	basket	0.4628451	0.1748206	0.0691802	0.293154
XLOC_016128	guanine nucleotide exchange factor in mesoderm	0.3341141	0.1527793	0.0738292	0.4392774
XLOC_016141	protein kinase N	0.2373499	0.2430165	0.1008946	0.418739
XLOC_017203	notum	0.3291655	0.1048545	0.1481245	0.4178556
XLOC_017247	spineless	0.4755031	0.0788446	0.1077313	0.3379211
XLOC_017278	single-minded	0.1406642	0.018595	0.1136307	0.7271101

Table S5 Numbers of genes in each module, annotated and are used for GO analysis in the IvM.

	M1	M2	M3	M4	M5	M6	M7	M8	M9	M10	M11	M12	M13
							IvM male module						
All	599	393	348	346	304	281	249	245	227	163	162	134	66
Annotated	337	234	253	205	212	193	160	130	166	102	116	85	36
GO query	298	212	227	178	193	167	148	119	152	94	110	75	32
	F1	F2	F3	F4	F5	F6	F7	F8	F9	F10	F11	F12	
							IvM female module						
All	708	424	386	341	327	302	238	206	195	170	162	123	
Annotated	425	244	278	253	205	191	165	117	119	75	104	55	
GO query	366	221	251	225	192	175	149	109	109	69	93	42	

Table S6 Numbers of genes in each module, annotated and are used for GO analysis in the UvIM.

	M1	M2	M3	M4	M5	M6	M7	M8	M9	M10	M11	
	UvIM male module											
All	1637	1008	570	506	503	499	426	273	214	191	101	
Annotated	1126	552	376	408	280	338	200	180	116	154	86	
GO query	1009	452	325	359	250	308	179	167	98	144	81	
	F1	F2	F3	F4	F5	F6	F7	F8	F9	F10	F11	F12
	UvIM female module											
All	1056	1032	659	609	474	432	409	342	333	155	116	100
Annotated	709	558	353	451	235	289	319	271	228	132	98	56
GO query	663	457	286	408	205	261	299	251	199	123	96	51

Table S7 The top 50 genes among the module memberships (MM) calculated from co-expression network analysis in the IvM comparison.

Gene ID	Gene name	MM	*P*-value
	Male module M2		
XLOC_017554	NA	0.98442	2.97E-27
XLOC_006219	expansion	0.983663	6.62E-27
XLOC_000890	NA	0.978236	8.32E-25
XLOC_017312	NA	0.977413	1.56E-24
XLOC_003387	NA	0.976209	3.72E-24
XLOC_011952	dusky-like	0.970221	1.62E-22
XLOC_005359	NA	0.968699	3.73E-22
XLOC_015690	uncharacterized protein Dmel_CG33978	0.968595	3.94E-22
XLOC_005358	NA	0.968428	4.31E-22
XLOC_004703	uncharacterized protein Dmel_CG14892	0.968004	5.39E-22
XLOC_001409	NA	0.967213	8.11E-22
XLOC_004206	CG9249	0.966758	1.02E-21
XLOC_003386	cuticular protein 66D	0.966721	1.04E-21
XLOC_005751	peritrophin A	0.964818	2.64E-21
XLOC_017323	uncharacterized protein Dmel_CG32645	0.962132	9.03E-21
XLOC_012622	NA	0.961957	9.76E-21
XLOC_017126	NA	0.959553	2.71E-20
XLOC_001700	ChLD3	0.95739	6.47E-20
XLOC_005304	NA	0.957223	6.91E-20
XLOC_017529	uncharacterized protein Dmel_CG30172	0.955923	1.14E-19
XLOC_003593	NA	0.955696	1.24E-19
XLOC_016811	NA	0.95566	1.26E-19
XLOC_005365	NA	0.955634	1.27E-19
XLOC_011360	uncharacterized protein Dmel_CG12947	0.954332	2.05E-19
XLOC_000434	NA	0.953787	2.50E-19
XLOC_014517	CG4660	0.952393	4.10E-19
XLOC_017274	NA	0.952284	4.26E-19
XLOC_005360	NA	0.95227	4.28E-19
XLOC_001484	NA	0.951134	6.33E-19
XLOC_005313	NA	0.950774	7.15E-19
XLOC_007485	CG5966	0.950157	8.80E-19
XLOC_000903	ejaculatory bulb protein III	0.95008	9.03E-19
XLOC_013894	sulfotransferase 4	0.95003	9.18E-19
XLOC_006676	uncharacterized protein Dmel_CG43333	0.94961	1.05E-18
XLOC_008307	Cyp303a1	0.949577	1.07E-18
XLOC_000749	NA	0.949201	1.21E-18
XLOC_017075	mitochondrial ribosomal protein L15	0.949045	1.27E-18
XLOC_005800	uncharacterized protein Dmel_CG32645	0.948726	1.41E-18
XLOC_011125	TweedleE	0.948404	1.56E-18

XLOC_012624	NA	0.948212	1.66E-18
XLOC_001218	Ance-3	0.947999	1.78E-18
XLOC_013079	NA	0.947303	2.22E-18
XLOC_005311	uncharacterized protein Dmel_CG30438	0.946559	2.80E-18
XLOC_007690	skeletor	0.946204	3.12E-18
XLOC_010577	CG8665	0.945822	3.51E-18
XLOC_004520	NA	0.945703	3.64E-18
XLOC_003213	cuticular protein 49Aa	0.945654	3.70E-18
XLOC_015116	IRSp53	0.945268	4.16E-18
XLOC_005454	NA	0.944366	5.45E-18
XLOC_005455	NA	0.942329	9.89E-18
Male module M5			
XLOC_011806	scraps	0.949963	9.39E-19
XLOC_003733	CG5705	0.947965	1.80E-18
XLOC_003811	spalt major	0.934689	7.72E-17
XLOC_003718	spalt major	0.932144	1.45E-16
XLOC_007734	Sp1	0.920815	1.83E-15
XLOC_018670	NA	0.913606	7.61E-15
XLOC_012795	Cyclin-dependent kinase 1	0.913265	8.12E-15
XLOC_011805	scraps	0.910453	1.37E-14
XLOC_003719	spalt-related	0.910056	1.47E-14
XLOC_009712	Eb1	0.909013	1.77E-14
XLOC_009451	SP2353	0.908356	1.99E-14
XLOC_018082	alpha-Tubulin at 84B	0.907747	2.22E-14
XLOC_011358	B4	0.904525	3.88E-14
XLOC_016001	off-track	0.902906	5.09E-14
XLOC_013977	uncharacterized protein Dmel_CG13126	0.901365	6.58E-14
XLOC_007778	NA	0.900279	7.85E-14
XLOC_007779	NA	0.896269	1.49E-13
XLOC_018016	CG9302	0.890735	3.44E-13
XLOC_017035	PR/SET domain containing protein 7	0.883443	9.75E-13
XLOC_002104	outer segment 6	0.882374	1.13E-12
XLOC_011968	borealin-related	0.879941	1.57E-12
XLOC_000651	Salt-inducible kinase 2	0.877921	2.05E-12
XLOC_007589	dumpy	0.87354	3.60E-12
XLOC_002020	NA	0.871938	4.40E-12
XLOC_005340	CAP-D2 condensin subunit	0.870013	5.59E-12
XLOC_006062	NA	0.868488	6.73E-12
XLOC_007510	tenectin	0.868376	6.82E-12
XLOC_000209	disabled	0.866936	8.12E-12
XLOC_007511	tenectin	0.865924	9.16E-12
XLOC_000371	Kinesin-like protein at 3A	0.862517	1.36E-11
XLOC_015886	kinesin heavy chain	0.861163	1.59E-11
XLOC_014496	fat-spondin	-0.85957	1.91E-11

XLOC_003810	NA	0.859008	2.04E-11
XLOC_005922	mapmodulin	0.856211	2.78E-11
XLOC_010269	Actin-related protein 1	0.854979	3.18E-11
XLOC_003017	drumstick	0.854678	3.29E-11
XLOC_017473	hormone receptor-like in 39	0.853626	3.69E-11
XLOC_014002	NA	0.852525	4.15E-11
XLOC_015217	flare	0.850866	4.95E-11
XLOC_014946	uncharacterized protein Dmel_CG12880	0.850348	5.23E-11
XLOC_005799	trynity	0.847322	7.17E-11
XLOC_003644	SoxNeuro	0.844518	9.56E-11
XLOC_015384	CG7102	0.842834	1.13E-10
XLOC_009611	misato	0.840414	1.44E-10
XLOC_012865	connectin	0.838549	1.73E-10
XLOC_015101	amun	0.837223	1.96E-10
XLOC_018629	uncharacterized protein Dmel_CG43370	0.831852	3.26E-10
XLOC_014025	NA	0.829664	3.99E-10
XLOC_011675	focal adhesion kinase	0.826631	5.26E-10
XLOC_013993	uncharacterized protein Dmel_CG42673	0.824608	6.30E-10
Male module M8			
XLOC_017722	NA	0.958279	4.55E-20
XLOC_014894	NA	0.951621	5.36E-19
XLOC_012497	NA	0.941348	1.31E-17
XLOC_000023	NA	0.940837	1.51E-17
XLOC_013560	NA	0.937429	3.81E-17
XLOC_005410	rough deal	0.934197	8.74E-17
XLOC_017763	NA	0.933499	1.04E-16
XLOC_006246	Ranbp9	0.931938	1.52E-16
XLOC_009078	moleskin	0.931149	1.84E-16
XLOC_010125	NA	0.927455	4.35E-16
XLOC_005149	NA	0.922855	1.19E-15
XLOC_007137	NA	0.915239	5.58E-15
XLOC_005688	CG8519	0.912885	8.72E-15
XLOC_002707	NA	0.912742	8.96E-15
XLOC_018671	megator	0.911552	1.12E-14
XLOC_018674	arginine methyltransferase 7	0.910156	1.44E-14
XLOC_018659	Hpr1	0.909741	1.55E-14
XLOC_004975	lethal (2) k09022	0.893552	2.26E-13
XLOC_002844	similar to deadpan	0.892368	2.70E-13
XLOC_005495	engrailed	0.88844	4.82E-13
XLOC_009972	NA	0.885356	7.47E-13
XLOC_012838	pod1	0.885138	7.70E-13
XLOC_017046	NA	0.881003	1.36E-12
XLOC_005013	lethal (2) k09022	0.878321	1.94E-12
XLOC_003137	NA	0.87486	3.05E-12

XLOC_000552	lethal (1) 1Bi	0.874364	3.24E-12
XLOC_005479	NA	0.873221	3.75E-12
XLOC_007384	complex I intermediate-associated protein, 30 kDa	0.869664	5.83E-12
XLOC_011205	castor	0.869662	5.84E-12
XLOC_015381	sans fille	0.866654	8.39E-12
XLOC_017045	NA	0.865545	9.58E-12
XLOC_005660	CG7099	0.86324	1.26E-11
XLOC_017007	forkhead domain 96Ca	0.861578	1.52E-11
XLOC_001920	gustavus	0.860772	1.67E-11
XLOC_017675	NA	-0.85704	2.54E-11
XLOC_008987	synaptojanin	0.857026	2.54E-11
XLOC_005328	NA	0.85696	2.56E-11
XLOC_015409	gooseberry-neuro	0.856182	2.79E-11
XLOC_010030	NA	0.856089	2.82E-11
XLOC_017496	NA	0.854374	3.40E-11
XLOC_014521	REPTOR-binding partner	-0.85332	3.81E-11
XLOC_008945	uncharacterized protein Dmel_CG10347	0.853303	3.82E-11
XLOC_010958	NA	0.85293	3.97E-11
XLOC_014704	topoisomerase 2	0.852096	4.35E-11
XLOC_014730	NA	0.851839	4.47E-11
XLOC_018644	NA	0.850159	5.34E-11
XLOC_009034	nucleoporin 358kD	0.846819	7.56E-11
XLOC_004386	tollo	0.846517	7.79E-11
XLOC_011790	papilin	0.840449	1.43E-10
XLOC_001984	mini spindles	0.835632	2.29E-10
Female module F2			
XLOC_001508	NA	0.984008	4.61E-27
XLOC_003486	Tie-like receptor tyrosine kinase	0.982526	2.06E-26
XLOC_012620	NA	0.982132	3.00E-26
XLOC_013076	NA	0.977113	1.94E-24
XLOC_007758	NA	0.975156	7.71E-24
XLOC_013075	NA	0.971868	6.22E-23
XLOC_001378	Msr-110	0.971677	6.97E-23
XLOC_003679	cadherin 96Ca	0.971516	7.67E-23
XLOC_006254	Calcium-dependent secretion activator	0.968863	3.41E-22
XLOC_007493	uncharacterized protein Dmel_CG34355	0.967759	6.12E-22
XLOC_013009	transport and golgi organization 5	0.967352	7.55E-22
XLOC_003483	neurotrophin 1	0.965839	1.61E-21
XLOC_003484	NA	0.965645	1.77E-21
XLOC_005359	NA	0.96454	3.01E-21
XLOC_011966	uncharacterized protein Dmel_CG10657	0.963031	6.05E-21
XLOC_003487	I-kappaB kinase beta	0.962359	8.17E-21
XLOC_010083	CG7530	0.962214	8.71E-21
XLOC_010539	NA	0.961871	1.01E-20

XLOC_012622	NA	0.961752	1.07E-20
XLOC_014517	CG4660	0.960967	1.50E-20
XLOC_005338	Rab30	0.960904	1.54E-20
XLOC_012184	NA	0.960511	1.82E-20
XLOC_015663	uncharacterized protein Dmel_CG34038	0.960225	2.05E-20
XLOC_005381	uncharacterized protein Dmel_CG11409	0.95938	2.91E-20
XLOC_005753	Ccp84Ae	0.95761	5.94E-20
XLOC_002143	NA	0.95377	2.52E-19
XLOC_018477	methuselah-like 1	0.95355	2.72E-19
XLOC_011403	stranded at second	0.953228	3.06E-19
XLOC_005304	NA	0.952705	3.68E-19
XLOC_002508	lysophosphatidylcholine acyltransferase	0.952315	4.22E-19
XLOC_002916	NA	0.952125	4.50E-19
XLOC_010598	NA	0.951703	5.21E-19
XLOC_007295	CG1632	0.951579	5.44E-19
XLOC_003681	NA	0.948588	1.47E-18
XLOC_002992	uncharacterized protein Dmel_CG15629	0.948543	1.49E-18
XLOC_011249	spidey	0.948039	1.76E-18
XLOC_007383	laccase 2	0.947805	1.89E-18
XLOC_007294	CG1632	0.947454	2.11E-18
XLOC_017303	NA	0.946003	3.32E-18
XLOC_004703	uncharacterized protein Dmel_CG14892	0.945513	3.86E-18
XLOC_007430	osiris 7	0.944105	5.89E-18
XLOC_005454	NA	0.942403	9.68E-18
XLOC_004520	NA	0.941306	1.32E-17
XLOC_009047	NA	0.94114	1.39E-17
XLOC_018463	MICAL-like	0.936016	5.50E-17
XLOC_016641	NA	0.935718	5.94E-17
XLOC_015116	IRSp53	0.93538	6.48E-17
XLOC_002883	NA	0.934288	8.54E-17
XLOC_011831	CG8172	0.931212	1.82E-16
XLOC_004772	NA	0.930817	1.99E-16
Female module F4			
XLOC_005368	C901	0.978992	4.59E-25
XLOC_001873	multiple wing hairs	0.968443	4.27E-22
XLOC_008049	CG4702	0.962789	6.74E-21
XLOC_006344	mind the gap	0.959993	2.26E-20
XLOC_008256	NA	0.957556	6.06E-20
XLOC_003263	crinkled	0.956882	7.89E-20
XLOC_003461	CG4374	0.956399	9.49E-20
XLOC_015687	NA	0.956034	1.09E-19
XLOC_013780	CG5278	0.95414	2.20E-19
XLOC_013234	uncharacterized protein Dmel_CG31559	0.952604	3.81E-19
XLOC_017805	CG7896	0.951818	5.01E-19

XLOC_003313	uncharacterized protein Dmel_CG42674	0.950454	7.97E-19
XLOC_012304	NA	0.947453	2.12E-18
XLOC_013734	CG5278	0.947098	2.36E-18
XLOC_004395	uncharacterized protein Dmel_CG14880	0.944696	4.94E-18
XLOC_002983	CG4678	0.94436	5.46E-18
XLOC_013398	uncharacterized protein Dmel_CG30463	0.943387	7.28E-18
XLOC_000732	megalin	0.939878	1.97E-17
XLOC_007162	NA	0.938824	2.62E-17
XLOC_012302	midline fasciclin	0.936826	4.46E-17
XLOC_006614	uncharacterized protein Dmel_CG11966	0.935422	6.41E-17
XLOC_017545	CG7896	0.931604	1.65E-16
XLOC_001379	pipe	0.930905	1.95E-16
XLOC_001938	uncharacterized protein Dmel_CG42598	0.930367	2.22E-16
XLOC_001937	NA	0.928983	3.07E-16
XLOC_010565	Ror	0.927035	4.79E-16
XLOC_014671	CG5567	0.924559	8.28E-16
XLOC_009689	CG9503	0.923837	9.68E-16
XLOC_001759	CG3961	0.920887	1.80E-15
XLOC_005277	CG3108	0.91974	2.28E-15
XLOC_013958	CG9095	0.919694	2.31E-15
XLOC_007317	uncharacterized protein Dmel_CG15211	0.917121	3.86E-15
XLOC_004682	uninflatable	0.91559	5.21E-15
XLOC_004683	uninflatable	0.915528	5.27E-15
XLOC_005933	uncharacterized protein Dmel_CG42268	0.914875	5.98E-15
XLOC_017670	NA	0.914226	6.77E-15
XLOC_004684	uninflatable	0.913776	7.37E-15
XLOC_005934	NA	0.912955	8.61E-15
XLOC_007136	NA	0.911404	1.15E-14
XLOC_015191	uncharacterized protein Dmel_CG17264	0.911385	1.15E-14
XLOC_009607	CG2767	0.90882	1.83E-14
XLOC_013186	NA	0.907372	2.37E-14
XLOC_005380	dishevelled associated activator of morphogenesis	0.907181	2.45E-14
XLOC_011905	cypher	0.906707	2.66E-14
XLOC_007422	phosphodiesterase 6	0.905842	3.09E-14
XLOC_011673	uncharacterized protein Dmel_CG32354	0.905561	3.25E-14
XLOC_012032	notopleural	0.904716	3.75E-14
XLOC_006325	dusky-like	0.904266	4.05E-14
XLOC_017502	cadherin 99C	0.90269	5.28E-14
XLOC_005282	CG3655	0.90235	5.59E-14
	Female module F6		
XLOC_012795	Cyclin-dependent kinase 1	0.98306	1.22E-26
XLOC_011806	scraps	0.966173	1.37E-21
XLOC_011968	borealin-related	0.962968	6.22E-21
XLOC_013975	non-claret disjunctional	0.96035	1.95E-20

XLOC_000371	Kinesin-like protein at 3A	0.956953	7.67E-20
XLOC_003733	CG5705	0.95651	9.10E-20
XLOC_000651	Salt-inducible kinase 2	0.952677	3.71E-19
XLOC_011805	scraps	0.941813	1.15E-17
XLOC_009611	misato	0.935382	6.48E-17
XLOC_002589	nessun dorma	0.935068	7.02E-17
XLOC_016135	aurora B	0.932342	1.38E-16
XLOC_000659	no distributive disjunction	0.929728	2.58E-16
XLOC_006062	NA	0.928657	3.31E-16
XLOC_015126	CG5235	0.923822	9.71E-16
XLOC_009134	scabrous	0.918126	3.16E-15
XLOC_017211	fizzy	0.917702	3.44E-15
XLOC_014946	uncharacterized protein Dmel_CG12880	0.914737	6.14E-15
XLOC_012145	NA	0.914448	6.49E-15
XLOC_002020	NA	0.911784	1.07E-14
XLOC_005769	pavarotti	0.908753	1.86E-14
XLOC_018265	NA	0.908275	2.02E-14
XLOC_013977	uncharacterized protein Dmel_CG13126	0.906331	2.84E-14
XLOC_002844	similar to deadpan	0.898765	1.00E-13
XLOC_006225	uncharacterized protein Dmel_CG14984	0.89671	1.39E-13
XLOC_014024	NA	0.893606	2.24E-13
XLOC_005410	rough deal	0.893533	2.27E-13
XLOC_007695	CG7550	0.89119	3.22E-13
XLOC_014025	NA	0.888824	4.55E-13
XLOC_002993	NA	0.888162	5.01E-13
XLOC_005340	CAP-D2 condensin subunit	0.88527	7.56E-13
XLOC_017035	PR/SET domain containing protein 7	0.883276	9.97E-13
XLOC_003644	SoxNeuro	0.874831	3.06E-12
XLOC_001512	NA	0.869159	6.20E-12
XLOC_016001	off-track	0.868065	7.09E-12
XLOC_005688	CG8519	0.864578	1.07E-11
XLOC_007778	NA	0.863699	1.19E-11
XLOC_007564	NA	0.86062	1.70E-11
XLOC_013225	NA	0.858483	2.16E-11
XLOC_018670	NA	0.851651	4.56E-11
XLOC_005495	engrailed	0.846624	7.71E-11
XLOC_014305	CG3689	0.844259	9.81E-11
XLOC_003017	drumstick	0.841372	1.31E-10
XLOC_017047	NA	0.840703	1.40E-10
XLOC_001894	moira	0.836875	2.03E-10
XLOC_018671	megator	0.835229	2.38E-10
XLOC_000552	lethal (1) 1Bi	0.833188	2.88E-10
XLOC_014151	NA	0.833124	2.90E-10
XLOC_015384	CG7102	0.832119	3.18E-10

XLOC_014695	charlatan	0.831638	3.33E-10
XLOC_008203	senseless	0.826684	5.23E-10

Table S8 The top 50 genes among the module memberships (MM) calculated from co-expression network analysis in the UvIM comparison.

Gene ID	Gene name	MM	*P*-value
	Male module M1		
XLOC_009752	Retinoblastoma-family protein	0.967247	7.97E-22
XLOC_016916	CENP-meta	0.966737	1.03E-21
XLOC_013560	NA	0.963637	4.59E-21
XLOC_012271	veloren	0.961958	9.75E-21
XLOC_014260	Nipped-B	0.960542	1.80E-20
XLOC_000133	oocyte maintenance defects	0.959743	2.51E-20
XLOC_017722	NA	0.959613	2.65E-20
XLOC_003755	nucleoporin 214kD	0.95718	7.03E-20
XLOC_000028	uncharacterized protein Dmel_CG15439	0.956747	8.31E-20
XLOC_003127	Hermansky-Pudlak syndrome 4	0.954682	1.81E-19
XLOC_008206	CG3887	0.953176	3.11E-19
XLOC_013365	NA	0.95282	3.53E-19
XLOC_011994	CAS/CSE1 segregation protein	0.952759	3.61E-19
XLOC_001907	ubiquitin conjugating enzyme E2M	0.951305	5.97E-19
XLOC_004946	cleavage and polyadenylation specificity factor 160	0.950811	7.06E-19
XLOC_011330	NA	0.950222	8.61E-19
XLOC_014142	uncharacterized protein Dmel_CG13900	0.949923	9.51E-19
XLOC_018560	circadian trip	0.948865	1.35E-18
XLOC_007443	nucleoporin 107kD	0.945064	4.42E-18
XLOC_011396	uncharacterized protein Dmel_CG12263	0.944783	4.81E-18
XLOC_011229	NA	0.943551	6.94E-18
XLOC_007657	NA	0.94293	8.31E-18
XLOC_009407	uncharacterized protein Dmel_CG12299	0.941326	1.32E-17
XLOC_001274	alien	0.940634	1.60E-17
XLOC_010732	NA	0.940551	1.63E-17
XLOC_009617	uncharacterized protein Dmel_CG12413	0.939863	1.98E-17
XLOC_014894	NA	0.939572	2.14E-17
XLOC_005281	Rab40	0.939137	2.41E-17
XLOC_018674	arginine methyltransferase 7	0.938177	3.12E-17
XLOC_005410	rough deal	0.938071	3.21E-17
XLOC_005033	archipelago	0.937369	3.87E-17
XLOC_012276	proliferating cell nuclear antigen	0.937064	4.19E-17
XLOC_008999	transportin	0.936661	4.66E-17
XLOC_010741	NA	0.935676	6.01E-17
XLOC_015444	NA	0.935555	6.20E-17
XLOC_014948	Grip163	0.934948	7.23E-17
XLOC_007670	uncharacterized protein Dmel_CG14894	0.934765	7.58E-17
XLOC_016713	decapping protein 2	0.934757	7.59E-17
XLOC_010642	NA	0.934562	7.97E-17

XLOC_017264	MORF-related gene 15	0.934087	8.98E-17
XLOC_006242	NA	0.93325	1.11E-16
XLOC_014179	ubiquitin specific protease 16/45	0.933117	1.14E-16
XLOC_005149	NA	0.932278	1.40E-16
XLOC_003056	pavarotti	0.931904	1.54E-16
XLOC_004947	bloom syndrome helicase	0.931336	1.76E-16
XLOC_014153	mediator complex subunit 23	0.931028	1.90E-16
XLOC_015218	Actin-related protein 5	0.930922	1.95E-16
XLOC_005837	uncharacterized protein Dmel_CG10803	0.93069	2.06E-16
XLOC_007449	NADH dehydrogenase (ubiquinone) B14 subunit	0.930501	2.15E-16
XLOC_002114	ankyrin repeat and LEM domain containing 2	0.930268	2.27E-16
	Male module M3		
XLOC_007690	Skeletor	0.987616	6.14E-29
XLOC_001700	ChLD3	0.976271	3.56E-24
XLOC_015690	uncharacterized protein Dmel_CG33978	0.972452	4.38E-23
XLOC_004167	uncharacterized protein Dmel_CG32816	0.970586	1.31E-22
XLOC_012622	NA	0.961949	9.79E-21
XLOC_003670	NA	0.960887	1.55E-20
XLOC_017312	NA	0.960403	1.90E-20
XLOC_018568	furrowed	0.958814	3.67E-20
XLOC_004534	NA	0.957928	5.24E-20
XLOC_003674	NA	0.957836	5.43E-20
XLOC_004772	NA	0.957172	7.05E-20
XLOC_015347	pipsqueak	0.956761	8.26E-20
XLOC_007493	uncharacterized protein Dmel_CG34355	0.955477	1.35E-19
XLOC_007383	laccase 2	0.955302	1.44E-19
XLOC_003911	uncharacterized protein Dmel_CG44098	0.955115	1.54E-19
XLOC_017554	NA	0.954186	2.17E-19
XLOC_014517	CG4660	0.953336	2.94E-19
XLOC_001796	uncharacterized protein Dmel_CG13634	0.951181	6.23E-19
XLOC_001378	Msr-110	0.951108	6.39E-19
XLOC_003671	NA	0.949916	9.53E-19
XLOC_003912	CG8654	0.949885	9.63E-19
XLOC_001508	NA	0.949551	1.08E-18
XLOC_006219	expansion	0.949384	1.14E-18
XLOC_001408	cuticular protein 56F	0.948078	1.73E-18
XLOC_015689	uncharacterized protein Dmel_CG33978	0.946814	2.59E-18
XLOC_004703	uncharacterized protein Dmel_CG14892	0.94547	3.91E-18
XLOC_001484	NA	0.944468	5.29E-18
XLOC_017303	NA	0.943704	6.63E-18
XLOC_017749	NA	0.942071	1.06E-17
XLOC_001196	NA	0.941392	1.29E-17
XLOC_015640	NA	0.941135	1.39E-17
XLOC_002303	hormone receptor-like in 38	0.940174	1.81E-17

XLOC_003387	NA	0.938809	2.63E-17
XLOC_002143	NA	0.936948	4.32E-17
XLOC_004114	hormone receptor 4	0.936121	5.36E-17
XLOC_012727	myosin heavy chain-like	0.936061	5.44E-17
XLOC_001773	NA	0.9354	6.45E-17
XLOC_011966	uncharacterized protein Dmel_CG10657	0.935229	6.73E-17
XLOC_004852	uncharacterized protein Dmel_CG14892	0.932432	1.35E-16
XLOC_011952	dusky-like	0.9322	1.43E-16
XLOC_001485	NA	0.930931	1.94E-16
XLOC_011831	CG8172	0.928347	3.55E-16
XLOC_004520	NA	0.927801	4.02E-16
XLOC_000903	ejaculatory bulb protein III	0.9264	5.52E-16
XLOC_000890	NA	0.925772	6.35E-16
XLOC_015688	NA	0.925297	7.05E-16
XLOC_003487	I-kappaB kinase beta	0.925104	7.35E-16
XLOC_017275	NA	0.924788	7.88E-16
XLOC_001197	NA	0.924772	7.90E-16
XLOC_013483	alternative testis transcripts open reading frame A	0.923932	9.48E-16
	Male module M5		
XLOC_016287	absent MD neurons and olfactory sensilla	0.970992	1.04E-22
XLOC_011673	uncharacterized protein Dmel_CG32354	0.968744	3.64E-22
XLOC_002661	NA	0.965238	2.16E-21
XLOC_014432	NA	0.956203	1.02E-19
XLOC_011880	chitinase 2	0.954429	1.98E-19
XLOC_011830	notopleural	0.953494	2.78E-19
XLOC_005218	NA	0.952738	3.63E-19
XLOC_014210	cyclin B	0.952721	3.66E-19
XLOC_007099	rotated abdomen	0.949568	1.07E-18
XLOC_011730	CG3587	0.945498	3.88E-18
XLOC_014169	NA	0.943748	6.54E-18
XLOC_008125	NA	0.942365	9.78E-18
XLOC_004860	serpin 28Dc	0.941828	1.14E-17
XLOC_012108	NA	0.939018	2.49E-17
XLOC_004152	NA	0.938127	3.16E-17
XLOC_007009	NA	0.932368	1.37E-16
XLOC_002664	NA	0.927558	4.25E-16
XLOC_017804	CG7896	0.926944	4.89E-16
XLOC_018477	methuselah-like 1	0.92645	5.46E-16
XLOC_004686	uninflatable	0.92488	7.72E-16
XLOC_014213	NA	0.923127	1.13E-15
XLOC_002663	NA	0.922974	1.16E-15
XLOC_012201	NA	0.919055	2.63E-15
XLOC_012037	NA	0.918532	2.92E-15
XLOC_007421	no mechanoreceptor potential C	0.918099	3.18E-15

XLOC_018513	NA	0.918	3.24E-15
XLOC_018077	Dpr-interacting protein beta	0.91756	3.54E-15
XLOC_004588	NA	0.917179	3.82E-15
XLOC_014224	NA	0.917139	3.85E-15
XLOC_012028	NA	0.917001	3.96E-15
XLOC_017545	CG7896	0.915439	5.36E-15
XLOC_016738	NA	0.91528	5.53E-15
XLOC_001379	pipe	0.914714	6.17E-15
XLOC_003723	uncharacterized protein Dmel_CG16798	0.914433	6.51E-15
XLOC_001074	TWIK-related acid-sensitive K[+] channel 7	0.913899	7.20E-15
XLOC_007068	NA	0.913192	8.23E-15
XLOC_014106	NA	0.913003	8.53E-15
XLOC_009732	patj	0.908659	1.89E-14
XLOC_012030	NA	0.908302	2.01E-14
XLOC_003722	smog	0.908168	2.06E-14
XLOC_016541	NA	0.906409	2.80E-14
XLOC_017806	CG7896	0.906222	2.90E-14
XLOC_005264	uncharacterized protein Dmel_CG10280	0.904048	4.20E-14
XLOC_015147	NA	0.902259	5.67E-14
XLOC_009359	nahoda	0.902162	5.77E-14
XLOC_018059	NA	0.90205	5.87E-14
XLOC_011439	uncharacterized protein Dmel_CG42326	0.901925	6.00E-14
XLOC_010437	CG3262	0.900083	8.11E-14
XLOC_002983	CG4678	0.898994	9.67E-14
XLOC_015037	NA	0.897924	1.15E-13
Male module M10			
XLOC_002139	Nucleosome-destabilizing factor	0.957609	5.94E-20
XLOC_004155	Rho GTPase activating protein at 54D	0.956641	8.65E-20
XLOC_008957	olf413	0.955036	1.59E-19
XLOC_017035	PR/SET domain containing protein 7	0.954283	2.09E-19
XLOC_012616	flapwing	0.951417	5.75E-19
XLOC_003832	Ras oncogene at 85D	0.947857	1.86E-18
XLOC_011805	scraps	0.938801	2.64E-17
XLOC_011968	borealin-related	0.935044	7.06E-17
XLOC_011806	scraps	0.934912	7.30E-17
XLOC_012562	CG2247	0.93044	2.18E-16
XLOC_011126	Eph receptor tyrosine kinase	0.927933	3.91E-16
XLOC_016154	epidermal growth factor receptor	0.925698	6.45E-16
XLOC_000371	Kinesin-like protein at 3A	0.924796	7.86E-16
XLOC_005340	CAP-D2 condensin subunit	0.921074	1.74E-15
XLOC_002104	outer segment 6	0.920988	1.77E-15
XLOC_000922	heterogeneous nuclear ribonucleoprotein K	0.913199	8.22E-15
XLOC_000651	Salt-inducible kinase 2	0.911336	1.16E-14
XLOC_017027	splicing factor 2	0.910958	1.25E-14

XLOC_007589	dumpy	0.909955	1.50E-14
XLOC_015217	flare	0.909154	1.73E-14
XLOC_003811	spalt major	0.908776	1.85E-14
XLOC_002154	neurotactin	0.908607	1.90E-14
XLOC_007734	Sp1	0.907461	2.33E-14
XLOC_015219	NA	0.905927	3.05E-14
XLOC_006613	ebi	0.905746	3.15E-14
XLOC_009492	doublesex	0.904773	3.72E-14
XLOC_002020	NA	0.904576	3.84E-14
XLOC_007483	distracted	0.903875	4.33E-14
XLOC_009712	Eb1	0.903854	4.34E-14
XLOC_009524	Srp54	0.903784	4.40E-14
XLOC_013976	NA	0.90318	4.87E-14
XLOC_009451	SP2353	0.903054	4.97E-14
XLOC_016155	epidermal growth factor receptor	0.901889	6.03E-14
XLOC_016001	off-track	0.900929	7.06E-14
XLOC_006061	GUK-holder	0.898552	1.04E-13
XLOC_015886	kinesin heavy chain	0.896293	1.48E-13
XLOC_012795	Cyclin-dependent kinase 1	0.89424	2.03E-13
XLOC_012373	serrano	0.893429	2.30E-13
XLOC_003718	spalt major	0.89168	2.99E-13
XLOC_018016	CG9302	0.88856	4.73E-13
XLOC_007368	Rfx	0.888528	4.75E-13
XLOC_009702	uncharacterized protein Dmel_CG11980	0.886905	6.00E-13
XLOC_011358	B4	0.886733	6.15E-13
XLOC_009738	simjang	0.880735	1.41E-12
XLOC_014182	stem cell tumor	0.880101	1.53E-12
XLOC_007456	uncharacterized protein Dmel_CG10217	0.880031	1.55E-12
XLOC_007243	protein phosphatase 2A at 29B	0.875718	2.73E-12
XLOC_016005	sticks and stones	0.871651	4.56E-12
XLOC_011708	uncharacterized protein Dmel_CG10466	0.868363	6.83E-12
XLOC_018670	NA	0.867182	7.88E-12
Female module F2			
XLOC_013636	NA	0.966918	9.42E-22
XLOC_001546	NA	0.95522	1.48E-19
XLOC_009247	NA	0.951263	6.06E-19
XLOC_001577	NA	0.945868	3.46E-18
XLOC_012894	NA	0.943373	7.31E-18
XLOC_004329	NA	0.941643	1.20E-17
XLOC_017890	Cyclin-dependent kinase 9	0.940783	1.53E-17
XLOC_018501	NA	0.937317	3.92E-17
XLOC_018469	CG7058	0.936986	4.28E-17
XLOC_000018	NA	0.9338	9.65E-17
XLOC_017614	NA	0.932432	1.35E-16

XLOC_006455	NA	0.931557	1.67E-16
XLOC_008486	uncharacterized protein Dmel_CG32767	0.930271	2.27E-16
XLOC_013614	NA	0.930185	2.32E-16
XLOC_014841	NA	0.929283	2.86E-16
XLOC_009258	NA	0.927478	4.33E-16
XLOC_002157	NA	0.92732	4.49E-16
XLOC_014439	T-complex chaperonin 5	0.92612	5.87E-16
XLOC_018451	kugelei	0.922554	1.27E-15
XLOC_001538	phosphatidylinositol 5-phosphate 4-kinase	0.922042	1.42E-15
XLOC_008324	NA	0.920581	1.92E-15
XLOC_009290	CG3776	0.919523	2.39E-15
XLOC_004019	deflated	0.918474	2.95E-15
XLOC_007075	topoisomerase 1	0.917196	3.81E-15
XLOC_006456	RNA polymerase II 140kD subunit	0.91472	6.16E-15
XLOC_002632	uncharacterized protein Dmel_CG11030	0.914519	6.40E-15
XLOC_013613	NA	0.913999	7.07E-15
XLOC_014607	NA	0.913744	7.42E-15
XLOC_007791	slingshot	0.912962	8.60E-15
XLOC_009297	Rabenosyn-5	0.910748	1.29E-14
XLOC_007787	NA	0.909378	1.66E-14
XLOC_002324	NA	0.908757	1.85E-14
XLOC_012954	mitochondrial ribosomal protein L34	0.908176	2.06E-14
XLOC_007228	RNA and export factor binding protein 1	0.907879	2.17E-14
XLOC_009291	Ras-related protein interacting with calmodulin	0.90591	3.06E-14
XLOC_014434	NA	0.904097	4.17E-14
XLOC_000965	dynein heavy chain 64C	0.902383	5.56E-14
XLOC_001131	NA	0.900971	7.02E-14
XLOC_016483	NA	0.89946	8.97E-14
XLOC_000967	dynein heavy chain 64C	0.899389	9.07E-14
XLOC_012930	CG3337	0.899361	9.11E-14
XLOC_013612	NA	0.898792	9.98E-14
XLOC_009231	CG2656	0.8981	1.11E-13
XLOC_013164	phosphatidylinositol glycan anchor biosynthesis, class S	0.895861	1.58E-13
XLOC_014125	NA	0.895431	1.69E-13
XLOC_005785	PCI domain-containing protein 2	0.89265	2.59E-13
XLOC_018816	NA	0.892176	2.78E-13
XLOC_018489	kugelei	0.889874	3.91E-13
XLOC_001170	nuclear receptor coactivator 6	0.889324	4.24E-13
XLOC_014992	Ubr3 ubiquitin ligase	0.887442	5.56E-13
	Female module F3		
XLOC_001508	NA	0.981211	7.00E-26
XLOC_003484	NA	0.979722	2.53E-25
XLOC_003679	cadherin 96Ca	0.979478	3.09E-25
XLOC_012619	NA	0.979013	4.51E-25

XLOC_018477	methuselah-like 1	0.976941	2.20E-24
XLOC_007294	CG1632	0.974815	9.70E-24
XLOC_003486	Tie-like receptor tyrosine kinase	0.97372	1.98E-23
XLOC_004658	NA	0.972306	4.78E-23
XLOC_006254	Calcium-dependent secretion activator	0.972085	5.47E-23
XLOC_009359	nahoda	0.969822	2.02E-22
XLOC_015663	uncharacterized protein Dmel_CG34038	0.96973	2.13E-22
XLOC_003913	organic cation transporter 2	0.968922	3.31E-22
XLOC_004534	NA	0.967755	6.13E-22
XLOC_003487	I-kappaB kinase beta	0.96759	6.68E-22
XLOC_005753	Ccp84Ae	0.967541	6.86E-22
XLOC_009047	NA	0.967132	8.45E-22
XLOC_010083	CG7530	0.964723	2.76E-21
XLOC_002696	NA	0.963457	4.98E-21
XLOC_017305	NA	0.961182	1.37E-20
XLOC_001378	Msr-110	0.96098	1.49E-20
XLOC_014517	CG4660	0.959836	2.41E-20
XLOC_007758	NA	0.958171	4.75E-20
XLOC_013009	transport and golgi organization 5	0.957935	5.22E-20
XLOC_014822	NA	0.957303	6.69E-20
XLOC_017304	NA	0.95687	7.92E-20
XLOC_004815	NA	0.956065	1.08E-19
XLOC_016641	NA	0.955659	1.26E-19
XLOC_002508	lysophosphatidylcholine acyltransferase	0.955239	1.47E-19
XLOC_004703	uncharacterized protein Dmel_CG14892	0.954987	1.61E-19
XLOC_007010	CG6055	0.954711	1.79E-19
XLOC_003485	NA	0.954601	1.86E-19
XLOC_002664	NA	0.954183	2.17E-19
XLOC_014105	NA	0.953637	2.64E-19
XLOC_007295	CG1632	0.953027	3.28E-19
XLOC_012620	NA	0.952716	3.66E-19
XLOC_012030	NA	0.952319	4.21E-19
XLOC_017308	NA	0.95174	5.14E-19
XLOC_009932	nahoda	0.951384	5.81E-19
XLOC_004006	uncharacterized protein Dmel_CG11836	0.95138	5.82E-19
XLOC_004816	NA	0.948799	1.38E-18
XLOC_005453	NA	0.947916	1.83E-18
XLOC_001484	NA	0.947061	2.39E-18
XLOC_004520	NA	0.946832	2.57E-18
XLOC_014107	NA	0.946355	2.98E-18
XLOC_000844	convoluted	0.94601	3.32E-18
XLOC_013075	NA	0.945682	3.67E-18
XLOC_008729	NA	0.94539	4.01E-18
XLOC_010539	NA	0.945337	4.07E-18

XLOC_004852	uncharacterized protein Dmel_CG14892	0.944211	5.71E-18
XLOC_015690	uncharacterized protein Dmel_CG33978	0.942166	1.04E-17
	Female module F6		
XLOC_010310	unc-13	0.957819	5.47E-20
XLOC_013904	NA	0.955005	1.60E-19
XLOC_001687	uncharacterized protein Dmel_CG18549	0.95494	1.64E-19
XLOC_014266	geko	0.948707	1.42E-18
XLOC_012289	CG9932	0.945227	4.21E-18
XLOC_013853	syndapin	0.944844	4.72E-18
XLOC_006163	defective proboscis extension response 4	0.944347	5.48E-18
XLOC_017952	artichoke	0.94326	7.55E-18
XLOC_005017	NA	0.942209	1.02E-17
XLOC_013532	uncharacterized protein Dmel_CG33156	0.935326	6.57E-17
XLOC_014265	CG7330	0.93255	1.31E-16
XLOC_002389	protein kinase, cAMP-dependent, catalytic subunit 3	0.931672	1.63E-16
XLOC_009521	NA	0.930613	2.09E-16
XLOC_005830	malic enzyme b	0.929949	2.45E-16
XLOC_006632	Z band alternatively spliced PDZ-motif protein 66	0.928174	3.70E-16
XLOC_013881	uncharacterized protein Dmel_CG17127	0.927709	4.11E-16
XLOC_012847	uncharacterized protein Dmel_CG31871	0.927632	4.18E-16
XLOC_001744	NA	0.927559	4.25E-16
XLOC_010311	unc-13	0.926714	5.14E-16
XLOC_013944	CG7888	0.925158	7.26E-16
XLOC_013244	Nna1 carboxypeptidase	0.920824	1.83E-15
XLOC_004193	bicoid interacting protein 1	0.920818	1.83E-15
XLOC_009016	uncharacterized protein Dmel_CG14964	0.920247	2.06E-15
XLOC_012127	NA	0.918148	3.15E-15
XLOC_011897	secreted Wg-interacting molecule	0.913746	7.42E-15
XLOC_002145	uncharacterized protein Dmel_CG43386	0.913383	7.94E-15
XLOC_010482	unc-13	0.912745	8.95E-15
XLOC_008383	CG6903	0.912628	9.15E-15
XLOC_015357	uncharacterized protein Dmel_CG14275	0.911574	1.11E-14
XLOC_005749	obstructor-A	0.910873	1.27E-14
XLOC_002368	uncharacterized protein Dmel_CG10433	0.908313	2.01E-14
XLOC_013864	scavenger receptor acting in neural tissue and majority of rhodopsin is absent	0.907897	2.16E-14
XLOC_017029	phosphatidylinositol 4-kinase III alpha	0.906508	2.76E-14
XLOC_014513	NA	0.905265	3.42E-14
XLOC_002102	NA	0.905167	3.47E-14
XLOC_013665	CG6847	0.90399	4.24E-14
XLOC_007365	uncharacterized protein Dmel_CG13643	0.903833	4.36E-14
XLOC_004939	uncharacterized protein Dmel_CG10737	0.902902	5.10E-14
XLOC_002144	uncharacterized protein Dmel_CG15249	0.901766	6.16E-14
XLOC_011360	uncharacterized protein Dmel_CG12947	0.899773	8.53E-14
XLOC_003411	CG2016	0.899567	8.81E-14

XLOC_002886	NA	0.898823	9.93E-14
XLOC_001695	SET and MYND domain containing, arthropod-specific, member 1	0.898319	1.08E-13
XLOC_006859	CG4495	0.898152	1.11E-13
XLOC_015369	uncharacterized protein Dmel_CG14397	0.897265	1.27E-13
XLOC_002103	NA	0.896867	1.35E-13
XLOC_010481	NA	0.896839	1.36E-13
XLOC_014346	CG4270	0.896643	1.40E-13
XLOC_017523	salivary glands marred	0.895526	1.67E-13
XLOC_015267	iso glutaminyl cyclase	0.893829	2.17E-13
Female module F7			
XLOC_002139	Nucleosome-destabilizing factor	0.968629	3.87E-22
XLOC_000662	gluon	0.966613	1.10E-21
XLOC_012562	CG2247	0.957292	6.72E-20
XLOC_011805	scraps	0.955954	1.12E-19
XLOC_013975	non-claret disjunctional	0.954345	2.04E-19
XLOC_005340	CAP-D2 condensin subunit	0.952921	3.41E-19
XLOC_002589	nessun dorma	0.952869	3.47E-19
XLOC_012862	polypeptide GalNAc transferase 6	0.951091	6.42E-19
XLOC_012795	Cyclin-dependent kinase 1	0.949749	1.01E-18
XLOC_012269	CG7728	0.94962	1.05E-18
XLOC_017035	PR/SET domain containing protein 7	0.949105	1.24E-18
XLOC_011416	uncharacterized protein Dmel_CG31368	0.948824	1.36E-18
XLOC_013976	NA	0.947153	2.32E-18
XLOC_014160	CG5466	0.946532	2.82E-18
XLOC_013529	without children	0.941951	1.10E-17
XLOC_002283	minus	0.93874	2.68E-17
XLOC_009612	NA	0.937461	3.77E-17
XLOC_008251	dachs	0.937234	4.01E-17
XLOC_006061	GUK-holder	0.936784	4.51E-17
XLOC_000371	Kinesin-like protein at 3A	0.936735	4.57E-17
XLOC_010722	cyclin E	0.936727	4.58E-17
XLOC_011737	CG5726	0.936434	4.94E-17
XLOC_011708	uncharacterized protein Dmel_CG10466	0.935851	5.74E-17
XLOC_002020	NA	0.935271	6.66E-17
XLOC_010639	Kinesin-like protein at 61F	0.934031	9.11E-17
XLOC_013537	chromatin assembly factor 1, p55 subunit	0.932874	1.21E-16
XLOC_002986	uncharacterized protein Dmel_CG30389	0.932569	1.31E-16
XLOC_001512	NA	0.932266	1.41E-16
XLOC_008230	sloppy paired 1	0.931235	1.81E-16
XLOC_011126	Eph receptor tyrosine kinase	0.929197	2.92E-16
XLOC_001675	structure specific recognition protein	0.926679	5.19E-16
XLOC_000651	Salt-inducible kinase 2	0.926233	5.73E-16
XLOC_000663	subito	0.925688	6.46E-16
XLOC_015219	NA	0.924873	7.73E-16

XLOC_003832	Ras oncogene at 85D	0.923863	9.63E-16
XLOC_016001	off-track	0.922502	1.29E-15
XLOC_003890	minichromosome maintenance 3	0.921655	1.54E-15
XLOC_017509	semaphorin 2b	0.921382	1.63E-15
XLOC_018671	megator	0.920603	1.91E-15
XLOC_002971	brother of odd with entrails limited	0.920001	2.17E-15
XLOC_003600	ribonucleoside diphosphate reductase small subunit	0.919382	2.46E-15
XLOC_006618	E2F transcription factor 1	0.919001	2.65E-15
XLOC_004155	Rho GTPase activating protein at 54D	0.917937	3.29E-15
XLOC_010711	NA	0.91763	3.49E-15
XLOC_011968	borealin-related	0.917446	3.62E-15
XLOC_007695	CG7550	0.916807	4.11E-15
XLOC_005769	pavarotti	0.916666	4.23E-15
XLOC_002844	similar to deadpan	0.91626	4.57E-15
XLOC_005632	GUK-holder	0.915937	4.87E-15
XLOC_006613	ebi	0.914488	6.44E-15
