# Supplementary material for: Role of Sex-Concordant Gene Expression in the Coevolution of Exaggerated Male and Female Genitalia in a Beetle Group
Source: Mol Biol Evol. 2021 Apr 27;38(9):3593–605. doi: 10.1093/molbev/msab122 (PMC8382896; doi:10.1093/molbev/msab122)
Supplement: msab122_Supplementary_Data [file msab122_supplementary_data.zip › response_to_reviwers210323.docx]

**Response to reviewer comments**

Thank you very much for reviewing our manuscript and giving useful comments and suggestions. We considered all of them carefully and revised the manuscript as described below.

Our response is described following >>>

In the manuscript text file, changed parts are indicated by blue letters.

Editors’ comments to the author:

Thank you for submitting your work for consideration. It has now been seen by two reviewers, whose comments appear below. You will see that both appreciated the attention to an important question in biology (sexual dimorphism and its evolution), but raised concerns about some elements of the study, with those of reviewer 2 being greater than those of reviewer 1. I personally agree with the concerns about reliability of the network inferences using data from Drosophila and how alignment of all three species reads to a single species' genome might impact the results, but would like to give the authors a chance to revise this work in light of the questions raised and then reconsider. I'll also note that although I agree with reviewer 2 about the types of experiments that would strengthen this work, I understand they are likely not feasible in this system. I will also say that I find it very surprising that the differences in expression between sexes were not greater, especially compared to the interspecific differences, so anything that can be done to strengthen this finding (perhaps including independent validation of inferred expression differences for at least some genes with an independent method not reliant upon genome alignments) would strengthen this work.

>>> We appreciate your kind review and helpful comments on our manuscript. Although we couldn’t add new experimental data, we reconsidered the framework of gene network analysis and revised the manuscript extensively. In response to the reviewers’ concern about reliability of the network inference based on PPI with Drosophila data, we abandoned use of PPI, performed WGCNA separately for males and females and estimated the hub genes based on module membership values. With regard to your comment on alternative way to estimate gene expression profiles not reliant upon genome alignments on one of three species, we considered use of quantitative real-time PCR for candidate genes, but it was not feasible due to shortage of remaining RNA samples. However, the expression variance partitioning is made for all the data combined, and the fairly small variance explained by sex compared to that by stage and species is not necessarily surprising as developmental genes usually show stage-specific expression and we picked up interspecific DEGs. Meanwhile, although the expression variation due to sex difference was small on average, there were genes showing large expression variation between the sexes, and some of these were genes for imaginal disc development and involved in genital morphogenesis, which might be related to sex-specific genital morphogenesis. We have reported related results in our previous study on *Carabus maiyasanus* (Nomura et al. 2020). In the revised manuscript, we made clear the meaning and interpretation of gene expression variance and added the information of genes involved. Although we used *C. uenoi* genome as the reference because it was the only draft genome among the three species, we could map all data on the reference genome at similar rates (table S1), and bias would be minimal.

Reviewer(s)' comments to author:


Reviewer: 1


Comments to the Author
The authors perform a large transcriptome sequencing study to test a hypothesis about the genetic basis of coevolved reproductive phenotypes in male and female ground beetles.  Species within the genus Carabus (Ohomopterus) have dramatically divergent morphology in male and female primary genital structures, and there is strong correlation in the morphology between the sexes within each species.  Mismatch between male and female morphologies results in several deleterious consequences during mating, which raises an important question in evolutionary genetics: How can seemingly non-homologous structures evolve in concert within species, but diverge so rapidly between species?  The authors posit that one mechanism capable of achieving this sex-specific coevolution-- and species divergence-- is the coexpression of genes important for development of these structures in both males and females.  They sequenced the transcriptomes from the segment primordia from which the terminalia develop in males and females from three different species across four different developmental time points during which genital morphogenesis occurs.  The comparison of these datasets reveals that gene expression variance between males and females within a species is significantly smaller than developmental stage and species differences.  Estimates of the gene co-expression network structure further suggest that limited differences exist between males and females.  

The genetic basis of coevolved phenotypes has long interested evolutionary biologists.  In particular, with respect to reproductive traits that are involved in intromission, it has been unclear how the effects of natural selection and sexual selection could act with opposite effects to both maintain function and promote morphological divergence among populations and ultimately species.  The results of this study make an important contribution toward understanding the genetic basis underlying the evolution of coevolved reproductive phenotypes.  The experiments described in this manuscript are well conceived, well performed, and well analyzed.  I will offer only a handful of suggestions that the authors might consider and include in their presentation:

>>> Thanks for reviewing our manuscript and providing helpful comments.

1) In the first section of the Results, the transcriptome comparison is described as showing that the gene expression variance between the sexes is incredibly small.  Yet, the results described in Fig. 2 seem potentially at odds with this result.  In particular, compared to the species and stage expression differences, the sex differences may seem small, but it appears that the differences in expression between males and females across the developmental time course are not trivial (i.e., thousands of genes; Fig. 2B, C)?  If it is the case that this is a "scale" issue for the magnitude of the differential expression, I think that this could be made clearer in the text.

>>> Thanks for this comment. As you mentioned, the seemingly contradicting results would be a scale issue for the magnitude of the differential expression. The variance partitioning was made for all data, and the variance according to the stage was the largest and that to the sex smallest. For figures 2A and 2B at each developmental stage, interspecific DEGs were classified only by the interspecific difference in each sex. Although there are many DEGs between species detected in one sex but not in the other (indicated as male-only or female-only DEGs), these interspecific DEGs are not necessarily genes showing statistically significant differences in gene expression between the sexes and the magnitude of expression variation is mostly small. We noted this point in Results:

Notably, there were many interspecific DEGs differentially expressed only in one sex at each developmental stage (figs. 2 A, B), which seemingly contradicted the relatively small variance explained by the sex difference (figs. 2C, D). However, the expression variances were partitioned according to the overall gene expression data including the effects of stage, species, and sex, whereas the categorization of interspecific DEGs at each stage only considered the interspecific expression difference in each sex without accounting for the magnitude of differential expression between sexes.

2) Weighted co-expression network analysis:  It is a bit unclear how this analysis was performed with respect to male versus female networks.  My understanding from the text is that the networks are being derived here primarily from comparisons across species, but it seems informative to perform a similar set of analyses just between male and female transcriptome data within species across the time course?  Was this somehow incorporated into the analysis and I am missing something?

>>> We appreciated the reviewer’s comment and revised our gene network analysis using WGCNA. In our previous analyses, we performed WGCNA for the data including both sexes to detect common modules for genital morphogenesis in both sexes, but this might not be appropriate. In our reanalysis, we have performed the WGCNA with DEGs between species separately for males and females in both IvM and UvIM comparisons, and later the modules with hub genes were compared between the sexes.

3) PPI analysis:  I wonder if the authors might incorporate a test to determine whether the connectivity observed for the hub genes is greater than expected by chance?  For example, genes like dac will likely show PPIs with many other genes in the genome by virtue of its molecular function.  Perhaps the authors could consider performing a permutation analysis to randomly sample sets of genes from the genome and ask whether the modules that they identified from their transcriptome analysis show higher connectivity for their hub genes than expected by chance.  Also, I think the authors should mention that using the Drosophila PPI database here could identify many false positives/negatives for true interactions within this group of species.  If the genitalia genotype-phenotype map is rapidly evolving in these tissues, these gene interactions might also be rapidly evolving, so a protein-protein interaction identified in Drosophila whole-body samples, may not necessarily occur in Carabus genital primordia.  

>>>We appreciated the reviewer’s comment and decided not to use PPI analysis. In our revised analysis, we detected the hub genes by module membership values in WGCNA.

4) Given the results, the authors might say something about the nature of gene expression with respect to the conservation of development among species.  In other words, males and females within species have similar gene expression profiles across genital development, followed by species differences, followed by stage differences.  This seems to suggest that the gene expression program that directs the development of male and female genitalia are largely conserved across species.  Along these lines is it known when genital morphogenesis occurs in males and females in this species group?  If so, this would be interesting and important information to include in the DEG comparisons.

>>> A micro-CT study for the morphogenetic process in the genitalia of *C. iwawakianus* and *C. maiyasanus* (i.e., IvM comparison) during the pupal stage just has been published recently by another research group (Terada et al. 2021). This study shows that interspecific differences in the genital morphology (length differences) became obvious 4-6 days after pupation for male copulatory piece, and 6-8 days for female vaginal appendix. We discuss that differential expression profile of genes in male module M5 and female module F6 might result in the observed interspecific differences in the elongation timing of genital parts. Although micro-CT data for *C. uenoi* have not been published, we expect that elongation of genital parts in *C. uenoi* occurs much earlier than the other species, and this would be related to the differential gene expression in M1, M10 and F7 at PpL to PE in the UvIM comparison. We have added these discussions in the revised text.

5) If genome sequences exist for each of the three species studied here, why only map the transcriptome sequence reads to *C. uenoi*?  Why not obtain expression estimates by mapping to each species individually?  My guess is that mapping to one of the species was used to help normalize gene expression estimates across all comparisons, and if this is the case, it could be more clearly described in the text.

>>>As you have guessed, we used the *C. uenoi* genome for normalization, and it was the only draft genome available to us among the three *Ohomopterus* species. We described this reason in Results and Materials and Methods.

6) Does gene expression from the anal primordia (segment A11?) contribute disproportionately to the gene expression sex-similarity within species?  As the anal structures likely do not differ between the sexes morphologically, is there any way to computationally “subtract” these genes from the expression datasets?  

>>> The samples from third instar larvae (PpE and PpL) might contain tissues from A11, but those from pupae are only of genital tissues. We cannot dissect tissues from A11 separately and have no way to subtract the expressions of genes for anal structure.

Minor comments:

-- Because “expression patterns” is a rather broad term which could include spatial localization of transcripts within tissues, I might suggest using a more precise term such as “expression timing profiles” to describe the transcriptome data across the comparisons described in the manuscript.

>>>We agreed with this comment and changed the wording.

-- line 62:  I would also cite Mayer on pleiotropy (1963, Animal Species and Evolution), as this idea originated with him.

>>>Here we consider pleiotropic effects of genes affecting genital morphology of both sexes, whereas Mayr’s (1963) pleiotropic hypothesis concerned with genes affecting genital and general (external) morphological characters. So, it is inappropriate to cite this reference.

-- lines 73-75: I would suggest including more detail here.  Perhaps describe the data from Drosophila that show different segment primordia contribute disproportionately to male versus female structures.  In other words, the male versus female external genital structures are in fact not homologous because they derive primarily from different cell lineages (i.e., segment primordia) during development.  

>>> We added a comment on the developmental difference of male and female genitalia in *Drosophila* as follows: “Although male and female genitalia may not be completely homologous in many animals (e.g., male and female genitalia derive from different segments and tissues in insects; Sánchez and Guerrero 2001),”

-- lines 181-183:  Although the authors indicate this in the Discussion, this seems like a good place to inform the reader that this assumption is informed by what is known about genes involved in genital development in Drosophila.

>>> According to your suggestion, we revised this part as follows: “Among the GO terms, we focused on ‘imaginal disc’ terms related to wing development or leg morphogenesis, which shared genes with genital morphogenesis in *Drosophila* (Sánchez and Guerrero 2001), and ‘cuticle development’ which may also be related to the species-specific genital morphology in *Ohomopterus* (Fujisawa et al. 2019).”

-- line 307: As the PPI analysis results can only suggest a potential interaction, I would encourage the authors to describe this a bit more cautiously here,  “…we constructed PPI networks within the modules to confirm the potential interactions of the genes…”

>>> We have not used the PPI analysis in the revised manuscript.

-- lines 440-447: I think that it would be helpful to the reader to briefly summarize the number of individuals measured at each time point for each species.

>>> We have described the number of individuals more clearly in the next paragraph (after sex determination): “The total number of samples after the sex determination was 72 (i.e., 3 samples x 4 stages x 2 sexes x 3 species).”

-- line 457:  Is there a reference to include here that describes the length difference in dsx isoforms in this group?

>>> We cited our paper, Nomura et al. (2020), which described the length differences.

Reviewer: 2

Comments to the Author

Nomura et al. investigate the genetic networks governing correlated changes in male and female genital morphology between species. This is an important and timely question to be tackling with molecular and genomic approaches, as we currently have scant information on how dramatic phenotypes that correlate between males and females evolve. The authors perform RNA-seq on developing male and female genitalia of three species of beetles at multiple time points and find more differences in gene expression between species than between sexes. They also use computational methods to construct networks that may be associated with genital development and find that some well-connected genes exhibit correlated gene expression changes in their dataset. The authors conclude that correlated changes in genital morphology may be due in part to shared genetic networks between males and females. While this study may provide useful insights into the genetic basis of interspecific variation, the main claims of the manuscript are not sufficiently supported, and would greatly benefit from reframing the manuscript and improving the accessibility of its sequencing data.

>>> We appreciated the reviewer’s thoughtful and helpful comments. We have reconsidered the analytical approach, though we could not add data, and revised the manuscript extensively. Also, we have archived annotated gene list including expression data at the database (figshare).

The central claim of this paper is that changes in shared genetic networks between males and females result in correlated evolution of genital structures. To support this claim, the authors cite RNA-seq data that revealed more differentially expressed genes between species than between sexes; indeed, the authors state explicitly in the Discussion that “only a few DEGs are involved in differentiation of sex specific genitalia within a species,” and that “most DEGs between the species may contribute to their interspecific differences in genital morphology in both sexes through their co-expression in the same gene networks.”. However, these conclusions seem premature at the moment. First, a global gene expression analysis may obscure important changes in a few genes that independently modulate male and female traits. Second, gene expression measurements in homogenized tissues do not have the spatial or temporal resolution to discern genetic networks in the development of specific genital structures, which is necessary to demonstrate the plausibility of the argument. In order to support the conclusions as stated, the authors should demonstrate that multiple network components are expressed in the developing structures undergoing diversification using spatially resolved measurements – immunohistochemistry or in situ hybridization. Importantly, it seems like a good expectation from the outset that greater interspecies variation in gene expression would exist compared with variation between the sexes, especially in homogenized tissue containing multiple cell types. Because of its limitations, that observation alone is insufficient to either support or refute the hypothesis concerning genetic correlations between the sexes.

>>>We appreciate your concern. There are several studies that perform gene expression analysis using RNA-seq data to examine expression differences between the sexes, and indeed a number of genes with sex-specific expression have been found (for example Ledón-Rettig et al. 2017). Therefore, investigations for genes with sex-specific expression by gene expression analysis using RNA-seq are considered to have a certain level of detection power, although detailed expression differences may be obscured. However, we agree that we need further study to confirm the findings in this study by using spatially resolved measurements such as in situ hybridization at the time when interspecific differences occur in genital tissues; at present this method is not feasible in *Ohomopterus*.

Because the manuscript focuses on shared genetic networks, it is also important to consider the methods the authors use to construct these networks. The authors cluster their gene expression data into modules, annotate those modules using GO terms, and then construct “protein-protein interaction” networks using STRING, a computational tool that integrates data from multiple sources, including text mining, co-expression, and experiments. While all of these choices have caveats, it is difficult to assess the validity of these networks because the STRING tool is so permissive – are these networks based on verified experimental data, or other less convincing sources of information? Additionally, the authors note that these networks are largely based on annotations from Drosophila, and it is to be expected that some network rewiring has occurred over the ~300 million years since beetles and flies shared a common ancestor. Without more experimental validation of the spatial and temporal expression of network components, it is difficult to accept that the networks presented in this article represent true interactions within common cells, and therefore the conclusions concerning the gene networks underlying variation between species or sexes require additional support.

>>>We appreciated this comment and abandoned use of PPI analysis. Instead, we performed WGCNA in each sex and determined hub genes for modules (gene networks) for each sex.

The dramatic morphological differences the authors observe between species are nevertheless highly charismatic, and may yield future insights into the genetic basis of male/female diversification, which would be useful for the field. However, in order to appeal to researchers in other evolutionary systems, the expression data should be accessible and computationally tractable. We suggest that the authors construct a master table that includes expression data for each gene, along with annotations of homologous genes in other insects. That way, other researchers can quickly search for genes of interest. We also note that while we do not believe the stated conclusions of the paper are well-supported, we could imagine a revised manuscript that focuses solely on candidate genes underlying interspecific variation in genitalia. Within this frame, the methods for constructing networks and identifying hub genes need only be justified as a screen for identifying plausible candidates, which may obviate the methodological criticisms outlined above.

>>> Thanks for this constructive comment. We have prepared an Excel data file including predicted gene names and expression data, and have deposited at figshare for publication.
